# Subjective cognitive decline in individuals with isolated REM sleep behavior disorder

**DOI:** 10.1038/s41531-025-01161-2

**Published:** 2025-10-07

**Authors:** Anja Ophey, Sinah Röttgen, Christopher E. J. Doppler, Daniel Scharfenberg, Konstantin Kufer, Ezequiel Farrher, Gereon R. Fink, Michael Sommerauer, Elke Kalbe

**Affiliations:** 1 Medical Psychology | Neuropsychology and Gender Studies, Center for Neuropsychological Diagnostics and Intervention (CeNDI), Faculty of Medicine and University Hospital Cologne, University of Cologne, Cologne, Germany; 2 Cognitive Neuroscience, Institute for Neuroscience and Medicine (INM-3), Research Centre Juelich, Juelich, Germany; 3 Department of Neurology, Faculty of Medicine and University Hospital Cologne, University of Cologne, Cologne, Germany; 4 Center of Neurology, Department of Parkinson, Sleep and Movement Disorders, University of Bonn, Bonn, Germany; 5 German Centre for Neurodegenerative Diseases (DZNE), Bonn, Germany; 6 Institute for Neuroscience and Medicine - Imaging Core Facility (INM-ICF), Research Centre Juelich, Juelich, Germany; 7 Institute for Neuroscience and Medicine 4 (INM-4), Research Centre Juelich, Juelich, Germany

**Keywords:** Risk factors, Neurological manifestations, Dementia, Neurodegenerative diseases, Parkinson's disease, Cognitive ageing

## Abstract

Subjective cognitive decline (SCD) may constitute an early marker of mild cognitive impairment (MCI) in individuals with isolated REM sleep behavior disorder (iRBD). In this cross-sectional study, 80 individuals with iRBD were classified into iRBD with MCI (RBD.MCI), with SCD (RBD.SCD+), and without both (RBD.SCD–) based on neuropsychological testing and the Multi-SubCoDE questionnaire. The prevalence of SCD in iRBD was 36.3%, with predominance of the amnestic multi-domain SCD profile. RBD.SCD+ reported more severe depressive symptoms than RBD.SCD– and showed lower cognitive performance than RBD.SCD– in global cognition and attention & working memory. Magnetic resonance imaging analyses revealed lower grey matter volume in the left superior frontal gyrus for RBD.SCD+ than RBD.SCD–, which was associated with increased SCD-severity and lower global cognition. SCD without MCI in iRBD is associated with subtle cognitive deficits and structural brain changes. The prognostic value of SCD in iRBD should be further determined in longitudinal studies.

## Introduction

Isolated rapid eye movement (REM) sleep behavior disorder (iRBD) may indicate an early α-synucleinopathy with most affected individuals developing manifest Parkinson’s disease (PD) or dementia with Lewy bodies (DLB)^1^. Mild cognitive impairment (MCI) is the essential criterion for prodromal DLB^2^ and also a criterion in the research criteria for prodromal PD^3^. To date, a higher rate of cognitive decline in DLB-converters is the only reliable clinical marker differentiating between individuals with iRBD developing a Parkinsonism-first vs. dementia-first syndrome^1^. Cross-sectional meta-analytical evidence suggests that individuals with iRBD perform worse than healthy controls (HC) in all cognitive domains, particularly executive functions and memory^4^. Given the potential impact of cognitive decline on quality of life and independence of those affected, which simultaneously increases the burden on caregivers and the public healthcare system^5,6^, early identification of individuals at heightened risk for cognitive decline is of utmost importance. This would allow for timely prevention and the definition of target groups for early intervention in α-synucleinopathies.

Similar to advancements in the field of Alzheimer’s disease (AD)^7,8^, a three-stage system from *subjective cognitive decline* (SCD) to MCI to dementia is increasingly employed to describe cognitive decline in α-synucleinopathies^9–11^. MCI is characterized by objectively quantifiable impairment in at least one cognitive domain and, in contrast to dementia, largely preserved independent activities of daily living^2,12,13^. SCD may constitute a prodrome of MCI: The Subjective Cognitive Decline Initiative (SCD-I) defined SCD as self-perceived decline in cognitive functioning, unrelated to an acute event, together with demographically adjusted performance within normal range on standardized cognitive tests^7^. Even in the absence of objectively quantifiable cognitive decline by standard criteria, the presence of SCD seems to be associated with subtle cognitive changes both in AD^14,15^ and advanced α-synucleinopathies, e.g., PD^10,11^. Findings about structural and functional neural correlates of SCD are heterogeneous but include reduced grey matter (GM) volume, cortical thinning, and network alterations in (medio-)temporal, occipito-parietal, and frontal areas^16,17^. Notably, the presence of SCD in cognitively healthy individuals increases the risk of progression to MCI or dementia^11,18^.

Of note, there is considerable heterogeneity in terminology use within the SCD field. We support the use of the term SCD, in line with established conventions in the literature^7^. Even when based on cross-sectional data, the underlying core concept refers to participants’ perceived decline from a previous level, and it is considered the most neutral descriptor^7^. The alternative term *subjective cognitive impairment* neglects the temporal dimension of perceived change over time, instead focusing more on a status-quo comparison - such as to others of the same age - or on the perception of functional difficulties in everyday life. Alternatives such as *subjective cognitive complaints* and *concerns* are also common, but may carry more negative connotations in the medical context^7,10^.

SCD has rarely been studied concerning early α-synucleinopathies and has not systematically been evaluated in iRBD. Two studies report an association between non-motor features of prodromal PD (e.g., hyposmia, constipation, probable RBD) and the presence of SCD^19,20^. Furthermore, in our recent analysis of clinical iRBD subtypes, the late-onset, aggressive iRBD phenotype was associated with higher SCD scores than the more benign subtype^21^. Taken together, SCD may serve as an early prodromal marker preceding the onset of objectively quantifiable MCI in iRBD. SCD may contribute to our understanding of diverging disease trajectories leading either to Parkinsonism-first or dementia-first pathways, and supporting stratification of individuals for comprehensive biomarker characterization and clinical trials.

With the present work, we aim to cross-sectionally characterize the presentation of SCD in iRBD regarding its prevalence and domain-specific profiles, and to investigate objective correlates of SCD in individuals with iRBD regarding clinical characteristics, cognitive performance, and brain-structural changes. We assessed SCD with the Multiple Domain Subjective Cognitive Decline Evaluation (Multi-SubCoDE), a questionnaire previously used and validated in local research studies in PD and iRBD^21–23^, which we officially introduce in the current work. The Multi-SubCoDE assesses SCD in six cognitive domains in alignment with the SCD-I recommendations^7^. Based on Level-II neuropsychological testing and the Multi-SubCoDE, individuals with iRBD included in the CogTrAiL-RBD study^24^ were classified into three distinct categories: iRBD without SCD (RBD.SCD–), iRBD with SCD (RBD.SCD+), and iRBD with MCI (RBD.MCI). Additionally, a healthy control group (HC) was included. While the characterization and the analysis of brain-structural correlates of SCD follows an explorative approach, we hypothesize increasing non-motor symptoms (e.g., depressive symptoms) as well as a decline of cognitive performance from HC and RBD.SCD– to RBD.SCD+ to RBD.MCI.

## Results

### Characterization of subjective cognitive decline in iRBD

**Fig. 1:**
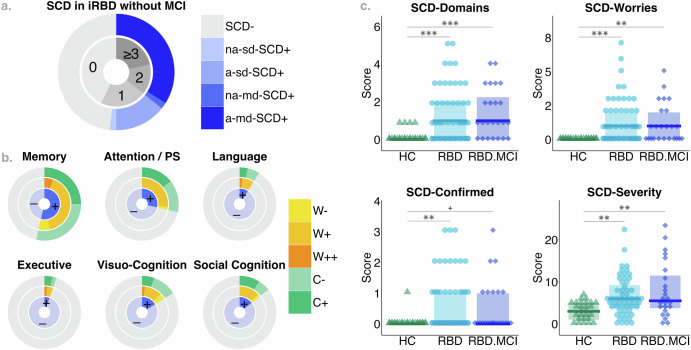
Characterization of subjective cognitive decline in individuals with iRBD without MCI. **a** Number of affirmed questions contributing to the Multi-SubCoDE SCD-Domains score and corresponding profile of subjective cognitive decline (SCD): SCD-, no SCD; na-sd-SCD+, non-amnestic single-domain SCD; a-sd-SCD+, amnestic single-domain SCD; na-md-SCD+, non-amnestic multi-domain SCD; a-md-SCD+, amnestic multi-domain SCD; iRBD, isolated REM sleep behavior disorder; MCI, mild cognitive impairment. **b** Domain-wise proportion of affirmed questions contributing to the Multi-SubCoDE SCD-Domains score and corresponding indication of worries (W-, no worries; W+, mild worries; W++, severe worries) and confirmation by an informant (C-, no; C+, yes). PS, processing speed. **c** Subscores of the Multi-SubCoDE across HC, RBD, and RBD.MCI groups. Triangles/dots/diamonds represent individual scores, the group-wise boxplots visualize the within-group median, and the hinges represent the corresponding first and third quartile. HC, healthy controls; RBD, individuals with iRBD without MCI, i.e., with and without SCD; RBD.MCI, individuals with iRBD with MCI. ^+^*p* < 0.100 *p < 0.050, **p < 0.010, ***p < 0.001.

Of *N* = 80 individuals with polysomnography-confirmed iRBD included in the present study, *n* = 29 (36.3%) were classified as RBD.SCD+ according to the criteria specified in the Methods section, while *n* = 24 (30%) exhibited objective MCI according to Level-II cognitive assessment. More than half of the individuals with iRBD without MCI affirmed impairment in at least one cognitive domain queried in the Multi-SubCoDE General questions (32/56, 57.1%, Fig. 1a). Of note, two individuals indicated SCD in the memory domain only and without accompanying worries, i.e., they did not fulfill the proposed criteria for RBD.SCD+. The questions on SCD in memory and attention/processing speed were the most frequently affirmed ones (Fig. 1b). The highest prevalence was observed for the amnestic multi-domain SCD+ (a-md-SCD+) profile (19/29, 65.5%), followed by the amnestic single-domain SCD+ (a-sd-SCD+) profile (8/29, 27.6%). Only one individual each classified with the non-amnestic single-domain SCD+ (na-sd-SCD+) profile (1/29, 3.4%) and one individual with the non-amnestic multi-domain SCD+ (na-md-SCD+) profile (1/29, 3.4%). Compared to HC, both individuals with and without MCI showed significantly higher scores in the SCD-Domains, SCD-Worries, SCD-Confirmed, and the SCD-Severity Multi-SubCoDE scores (Fig. 1c). Group differences were small to medium (0.30 ≤ |Cohen’s *d*| ≤ 0.53). Further sample characteristics and group comparisons are reported in Supplementary Table 1 and Supplementary Table 2.

### Correlates of subjective cognitive decline in iRBD

Sample characteristics of HC and individuals with RBD.SCD–, RBD.SCD+, and RBD.MCI as well as results of Analysis of Variance (ANOVA) and Analysis of Covariance (ANCOVA) models comparing these four groups are presented in Table 1. Full test statistics for each pairwise comparison are reported in Supplementary Table 3. There were no differences regarding age and sex distribution between groups. Overall, individuals with iRBD were 69.20 ± 5.93 years old, 12.5% (10/80) were female, and they reported a time of 9.60 ± 6.32 years since their first retrospectively reported RBD symptoms. Individuals with RBD.MCI reported significantly less years of total education than both HC and individuals with RBD.SCD– with small effect sizes (0.34 ≤ |Cohen’s *d*| ≤ 0.38).

**Table 1:** Sample characteristics

		HC *n* = 27	RBD.SCD– *n* = 27	RBD.SCD+ *n* = 29	RBD.MCI *n* = 24	Main effect group
Age *in years*		67.02 (4.86) [58.04–77.97]	70.92 (5.51) [57.63–80.63]	68.66 (6.29) [55.90–80.93]	67.92 (5.72) [59.15–79.02]	*F*(3,103) = 2.34, *p* = 0.078, _p_η^2^ = 0.06
Sex, n (%)	female	3 (11.11%)	6 (22.22%)	2 (6.90%)	2 (8.33%)	χ^2^(3) = 3.67, *p* = 0.299
	male	24 (88.89%)	21 (77.78%)	27 (93.10%)	22 (91.67%)	
Education *in years*		17.06 (3.54) [11.50–27.00]	16.80 (2.56) [13.00–22.00]	15.41 (3.11) [10.00–21.00]	14.71 (3.14) [7.00–21.00]	***F*(3,103)** = **3.36,** ***p*** = **0.022**^**c,e**^, _**p**_**η**^**2**^ = **0.09**
Time since first reported RBD symptoms *in years*		n.a.	10.32 (5.32) [1.92–20.52]	9.59 (7.17) [1.97–30.93]	8.80 (6.44) [2.39–27.34]	*F*(2,64) = 0.3, *p* = 0.744, _**p**_η^2^ = 0.01
RBDSQ total score		1.77 (1.37) [0–5]	8.78 (3.13) [0.00–12.00]	9.25 (2.63) [3.00–13.00]	8.26 (2.49) [2.00–12.00]	***F*(3,100)** = **52,** ***p*** < **0.001**^**a,b,c**^, _**p**_**η**^**2**^ = **0.61**
MDS-UPDRS-III total score		4.00 (3.93) [0–14]	4.94 (5.09) [0–19]	5.15 (4.36) [0–15]	5.09 (4.39) [1–17]	*F*(3,62) = 0.27, *p* = 0.849, _**p**_**η**^2^ = 0.01
Purdue Pegboard, dominant hand		–0.07 (0.85) [–1.93–1.38]	–0.74 (0.71) [–2.07–0.42]	–0.68 (0.84) [–3.25–1.15]	–1.08 (1.02) [–3.03–0.70]	***F*(3,103)** = **6.2,** ***p*** = **0.001**^**a,b,c**^, _**p**_**η**^**2**^ = **0.15**
BDI-II total score		1.41 (1.80) [0–7]	2.52 (2.72) [0.00–12.00]	8.55 (6.13) [1–28]	7.00 (6.67) [0–25]	***F*(3,103)** = **14.23,** ***p*** < **0.001**^**b,c,d,e**^, _**p**_**η**^**2**^ = **0.29**
NMSQ total score		2.15 (1.90) [0–8]	5.30 (2.63) [0–12]	7.62 (3.93) [2–15]	7.04 (4.24) [0–18]	***F*(3,103)** = **15.09,** ***p*** < **0.001**^**a,b,c,d,e**^, _**p**_**η**^**2**^ = **0.31**
NMSQ memory problems, Item 12, “yes”		2 (7.4%)	7 (25.9%)	14 (48.3%)	11 (45.8%)	**χ**^**2**^**(3)** = **13.835,** ***p*** = **0.003**
NMSQ attentional difficulties, Item 15, “yes”		1 (3.7%)	2 (7.4%)	12 (41.4%)	10 (41.7%)	**χ**^**2**^**(3)** = **19.474,** ***p*** < **0.001**
NMSQ anxiety, Item 17, “yes”		0 (0%)	1 (3.70%)	2 (6.90%)	2 (8.33%)	χ^2^(3) = 2.385, *p* = 0.496
MoCA total score*		27.26 (1.91) [24–30]	26.44 (1.99) [23–30]	26.76 (2.06) [23–30]	25.25 (1.82) [23–29]	***F*(3,102)** = **4.18,** ***p*** = **0.008**^**c,f**^, _**p**_**η**^**2**^ = **0.11**
Global cognition*		0.52 (0.21) [0.16–1.01]	0.59 (0.23) [0.13–1.18]	0.43 (0.28) [–0.01–1.23]	0.08 (0.31) [–0.44–0.74]	***F*(3,102)** = **16.96,** ***p*** < **0.001**^**c,d,e,f**^, _**p**_**η**^**2**^ = **0.33**
Executive functions*		0.49 (0.35) [–0.06–1.19]	0.59 (0.35) [0.02–1.20]	0.45 (0.41) [–0.63–1.25]	0.04 (0.60) [–1.19–1.42]	***F*(3,102)** = **7,** ***p*** < **0.001**^**c,e,f**^, _**p**_**η**^**2**^ = **0.17**
Attention & working memory*		0.69 (0.54) [–0.25–1.75]	0.71 (0.48) [–0.40–1.92]	0.53 (0.54) [–0.53–1.99]	0.22 (0.60) [–0.60–1.49]	***F*(3,102)** = **5.48,** ***p*** = **0.002**^**b,c,d,e,f**^, _**p**_**η**^**2**^ = **0.14**
Memory*		0.49 (0.61) [–0.69–1.50]	0.57 (0.45) [–0.18–1.39]	0.42 (0.55) [–0.69–1.68]	–0.22 (0.54) [–1.21–0.65]	***F*(3,102)** = **9.55,** ***p*** < **0.001**^**c,e,f**^, _**p**_**η**^**2**^ = **0.22**
Visuo-cognition*		0.66 (0.29) [–0.06–1.32]	0.67 (0.31) [0.05–1.32]	0.56 (0.38) [–0.38–1.27]	0.35 (0.47) [–0.38–1.37]	***F*(3,102)** = **4.73,** ***p*** = **0.004**^**c,e**^, _**p**_**η**^**2**^ = **0.12**
Language*		0.27 (0.26) [–0.25–0.71]	0.32 (0.27) [–0.25–0.85]	0.15 (0.40) [–0.74–1.06]	0.03 (0.36) [–0.83–0.55]	***F*(3,102)** = **3.13,** ***p*** = **0.029**^**c,e**^, _**p**_**η**^**2**^ = **0.08**
Social cognition*		–0.39 (1.17) [–2.33–2.33]	–0.45 (0.82) [–2.33–0.81]	–0.71 (0.99) [–2.33–1.88]	–0.82 (0.87) [–2.33–0.92]	*F*(3,102) = 0.39, *p* = 0.763, _**p**_**η**^2^ = 0.01

**Fig. 2:**
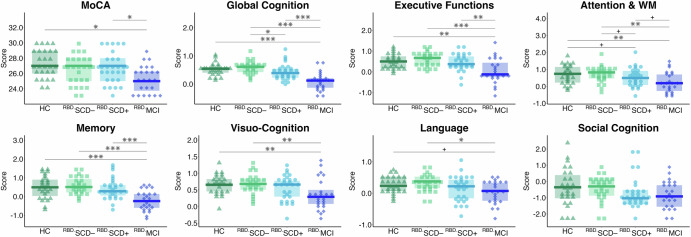
Global and domain-wise cognitive performance. Triangles/squares/dots/diamonds represent individual scores, the group-wise boxplots visualize the within-group median, and the hinges represent the corresponding first and third quartiles. HC, healthy controls; RBD.SCD–, individuals with iRBD without subjective cognitive decline; RBD.SCD+, individuals with iRBD with subjective cognitive decline; RBD.MCI, individuals with iRBD with mild cognitive impairment; WM, working memory. FDR-adjusted significance of paired-sample *t*-tests based on estimated marginal means from ANCOVA models adjusted for depressive symptoms is indicated as follows: ^+^<0.10, *p < 0.05, **p < 0.010, ***p < 0.001.

Data are mean (standard deviation) [range: minimum - maximum] unless indicated otherwise. The main effect of group (HC, RBD.SCD–, RBD.SCD+, and RBD.MCI) of an ANOVA model (or *ANCOVA model adjusted for depressive symptoms for cognitive scores) is reported. Significant models appear in bold. If *p* < 0.010, significant post hoc *t*-tests based on the estimated marginal means controlling the false discovery rate (FDR) across multiple comparisons are indicated as follows: ^a^HC vs. RBD.SCD–, ^b^HC vs. RBD.SCD+, ^c^HC vs. RBD.MCI, ^d^RBD.SCD– vs. RBD.SCD+, ^e^RBD.SCD– vs. RBD.MCI, ^f^RBD.SCD+ vs. RBD.MCI. For details, see Fig. 2 and Supplementary Table 3.

*BDI-II* Beck Depression Inventory, *HC* healthy controls, *MCI* mild cognitive impairment, *MDS-UPDRS-III* Movement Disorder Society Unified Parkinson’s Disease Rating Scale, *MoCA* Montréal Cognitive Assessment, *NMSQ* Non-Motor Symptoms Questionnaire, *RBD* REM sleep behavior disorder, *RBDSQ* REM Sleep Behavior Sisorder Screening Questionnaire, *SCD* subjective cognitive decline.

#### Clinical characteristics

ANOVAs revealed large group differences in depressive symptoms (Beck Depression Inventory, BDI-II, _p_η^2^ = 0.29) and overall non-motor symptoms (Non-Motor Symptoms Questionnaire, NMSQ, _p_η^2^ = 0.31). Descriptively, increasing depressive symptoms and non-motor symptoms were observed from HC and RBD.SCD– to RBD.SCD+ and RBD.MCI. As indicated by post hoc *t*-tests, the RBD.SCD+ and RBD.MCI groups reported more severe depressive symptoms than HC and RBD.SCD– with small to medium effect sizes (0.47 ≤ |Cohen’s *d*| ≤ 0.75). RBD.SCD+ and RBD.MCI reported more non-motor symptoms than RBD.SCD– with small effect sizes (0.26 |Cohen’s *d*| = 0.35). All iRBD groups reported more non-motor symptoms than HC with small to medium effect sizes (0.48 ≤ |Cohen’s *d*| ≤ 0.83). No significant group differences were observed in the dichotomous assessment of anxiety symptoms as measured by the NMSQ anxiety item. For fine motor abilities (Purdue Pegboard dominant hand, _p_η^2^ = 0.15), HC performed better than all iRBD groups with small to medium effect sizes (0.35 ≤ |Cohen’s *d*| ≤ 0.59). No meaningful group differences were observed in PD motor symptom severity (Movement Disorder Society Unified Parkinson’s Disease Rating Scale, MDS-UPDRS-III, _p_η^2^ = 0.01).

#### Cognition

ANCOVAs adjusted for depressive symptoms revealed medium to large group differences across all cognitive outcomes except social cognition (0.08 [language] ≤ _p_η^2^ ≤ 0.33 [global cognition]). Descriptively, cognitive performance was lowest in the RBD.MCI group, followed by the RBD.SCD+ group, and then the RBD.SCD– group across all cognitive domains (Fig. 2).

As indicated by post hoc *t*-tests based on the estimated marginal means (EMM) of the ANCOVA models, the RBD.SCD+ group showed lower cognitive performance compared to the RBD.SCD– group in global cognition (|Cohen’s *d*| = 0.31) with a small effect size. For the domain of attention & working memory, a trend toward statistical significance (*p*_FDR_ < 0.100) was observed for the RBD.SCD+ vs. RBD.SCD– comparison (|Cohen’s *d*| = 0.25). The global Multivariate Analysis of Covariance (MANCOVA) for the RBD.SCD– vs. RBD.SCD+ group comparison across cognitive outcomes failed statistical significance (for details, see Supplementary Table 3). The RBD.SCD+ group performed better than RBD.MCI in global cognition, executive functions, attention & working memory, and memory with medium effect sizes (0.26 [attention & working memory] ≤ |Cohen’s *d*| ≤ 0.64 [global cognition]), supported by a significant main effect of group in the global MANCOVA model. HC and the RBD.SCD– group showed better cognitive performance compared to the RBD.MCI group across all cognitive domains, except for social cognition. Differences were observed with small to medium effect sizes for HC (0.33 [language] ≤ |Cohen’s *d*| ≤ 0.79 [global cognition]) and medium to strong effect sizes for the RBD.SCD– group (0.41 [language] ≤ |Cohen’s *d*| ≤ 0.94 [global cognition]), supported by significant main effects of group in the global MANCOVA models for these comparisons.

#### Structural brain changes

**Fig. 3:**
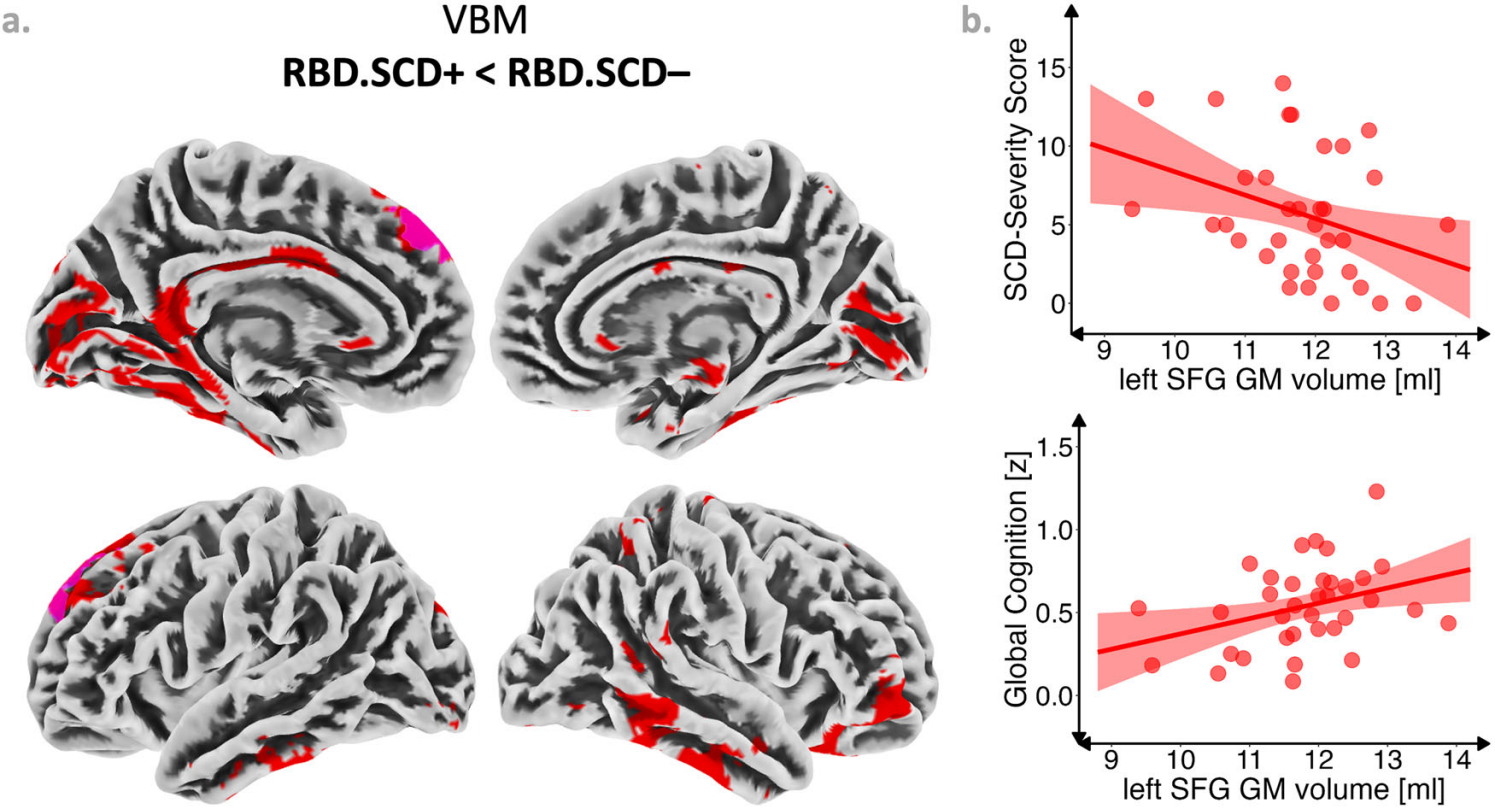
Structural brain changes and subjective cognitive decline. **a** Voxel-Based Morphometry (VBM) comparison between individuals with isolated REM sleep behavior disorder with (RBD.SCD+) and without (RBD.SCD–) subjective cognitive decline (SCD). Magenta indicates reduced grey matter (GM) volume in RBD.SCD+ compared to RBD.SCD– at *p*_FDR_ < 0.05 in parametric VBM analysis. Red highlights areas significant at *p* < 0.01 (uncorrected) following threshold-free cluster enhancement (TFCE). **b** Association between GM volume in the left superior frontal gyrus (SFG) and the SCD-Severity score of the Multiple Domain Subjective Cognitive Decline Evaluation (Multi-SubCoDE) and the global cognition composite score. Dots represent observed values; the line indicates the linear relationship between the two variables with 95% confidence interval.

T1-weighted brain images of *n* = 35 individuals with iRBD without MCI were acquired to investigate group differences between the RBD.SCD– and RBD.SCD+ group in GM volume (voxel-based morphometry, VBM) and cortical thickness (surface-based morphometry, SBM). Parametric VBM analysis controlled for age, depressive symptoms, and total intracranial volume revealed reduced GM volume in RBD.SCD+ (*n* = 16) compared to RBD.SCD– (*n* = 19) in a voxel cluster (k_E_ = 611 voxels, *p*_FDR_ = 0.011) peaking in the left superior frontal gyrus (SFG, peak Montréal Neurological Institute [MNI] coordinates *X*/*Y*/*Z* = –10/51/45). Non-parametric threshold-free cluster enhancement (TFCE) did not reveal suprathreshold clusters at FDR-corrected *p* < 0.05. Uncorrected TFCE results (*p* < 0.01) revealed reduced GM volume in RBD.SCD+ compared to RBD.SCD– in bilateral frontal, temporal, and parieto-occipital regions, including the left superior and middle frontal gyrus, the right orbitofrontal gyrus, the bilateral inferior and right middle temporal gyrus, the left fusiform and parahippocampal area and right hippocampus, and the bilateral cuneus and lingual gyrus (Fig. 3a). Whole-brain region-of-interest (ROI-)analyses confirmed structural changes from VBM voxel-wise analyses, particularly GM volume differences between RBD.SCD– and RBD.SCD+ for the left SFG, the only ROI surviving Holm–Bonferroni-correction at *p* < 0.05 (*T* = 3.20, *p*_Holm-Bonferroni_ = 0.038). Outside of this group comparison, lower GM volume in the left SFG was associated with increased SCD severity as measured with the Multi-SubCoDE SCD-Severity score (*r* = –0.33 [95%CI –0.64; –0.01]) and with lower cognitive performance as measured with the global cognition composite score (*r* = 0.34 [95%CI 0.01; 0.61]) (Fig. 3b). Neither parametric (voxel-wise and ROIs) nor non-parametric analyses revealed suprathreshold clusters at Holm–Bonferroni-corrected *p* < 0.05 (nor uncorrected *p* < 0.001) for cortical thickness compared between RBD.SCD+ and RBD.SCD–. Uncorrected ROI-analyses for both VBM and SBM are reported in Supplementary Table 4.

## Discussion

In this cross-sectional study, we characterized the presentation of SCD as an early marker of objective cognitive decline in iRBD. Our main results indicate that (i) the overall prevalence of SCD in individuals with iRBD was 36.3%, (ii) the amnestic multi-domain SCD profile was predominant, with memory, followed by attention/processing speed, being the most frequently affirmed SCD-domains, (iii) RBD.SCD+ reported more severe depressive symptoms than RBD.SCD– with a medium effect size, (iv) compared to RBD.SCD–, RBD.SCD+ showed lower cognitive performance with a small effect size in global cognition, potentially driven by the attention & working memory domain, (v) whole-brain VBM and whole-brain ROI-analyses revealed lower GM volume in the left SFG for RBD.SCD+ compared to RBD.SCD–, which correlated with more SCD and lower global cognition.

Since introducing the core research criteria for SCD in preclinical AD^7^, the field of SCD research has benefitted from guidelines for the operationalization, assessment, and reporting of SCD. Still, following AD tradition^25,26^, the memory domain is the most frequently –and often exclusively– assessed SCD domain in PD research^10,11^. With the Multi-SubCoDE, we introduced a questionnaire to assess multi-domain SCD in alignment with the SCD-I recommendations, e.g., regarding assessing SCD in memory and non-memory domains, worries, and the confirmation of cognitive decline by an informant^7^. The multi-domain assessment of SCD already appeared beneficial in advanced α-synucleinopathies. For instance, during multi-domain SCD assessments in manifest PD, SCD was reported for executive functions, attention, and language, but not memory^27,28^. Therefore, the Multi-SubCoDE may be particularly useful to assess SCD in (early) α-synucleinopathies.

The dominance of the amnestic SCD profiles and the low prevalence of SCD in the executive domain in our individuals with iRBD without MCI was unexpected, given the assumption that SCD domains reflect objective cognitive domains. Nevertheless, the multi-domain assessment of SCD in α-synucleinopathies appears advisable: Memory is, among executive functions, the most severely affected cognitive domain in iRBD, while impairments in executive functions have been identified as risk factors for conversion to advanced α-synucleinopathies^4^. Besides, cognitive profiles differ between AD and advanced α-synucleinopathies^29^: In manifest PD, it is primarily the non-amnestic domains that are particularly vulnerable and affected early^9^, and the non-amnestic MCI subtype is the most commonly observed^30^. Subjectively perceived cognitive performance does not necessarily reflect objective cognitive functioning. The observed “mismatch” regarding the attribution of subjectively observed changes to theoretically defined cognitive domains may indicate the disputable ecological validity of the domain view on cognition. Furthermore, cognitive problems in everyday situations are often misattributed to memory, as individuals tend to interpret most cognitive lapses in daily life as memory-related, rather than recognizing potential underlying attentional or executive deficits. Accordingly, the interpretation of SCD profiles (na-sd-SCD+, a-sd-SCD+, na-md-SCD+, a-md-SCD+), which were used to describe the pattern of SCD across domains in the present study, should be considered purely descriptive and interpreted with great caution. Future studies should aim to validate these profiles in large-scale samples by disentangling these domain-specific profiles with objective cognitive performance and evaluating their prognostic value. It may also be of interest to evaluate the correspondence between SCD profiles (and also MCI subtypes) in iRBD and underlying co-pathologies, such as Alzheimer’s disease or vascular disease^31^, as these may contribute to the observed patterns of domain-specific impairment. However, it has been shown that in general memory clinic populations, Lewy body pathology - rather than AD biomarkers - appears to be the primary driver of cognitive decline^32^.

Overall, our data support the notion of a continuum of cognitive decline along the spectrum from RBD.SCD– to RBD.SCD+ to RBD.MCI. These findings are in line with findings on SCD in manifest PD, where meta-analytical evidence revealed overall weak associations of SCD with cognitive changes on objective testing in cross-sectional studies across cognitive domains and moderate associations to neuropsychiatric symptoms such as anxiety and depression^11^. In our cohort, depressive symptoms were more severe in individuals with RBD.SCD+ and RBD.MCI compared to RBD.SCD–, supporting the close interaction between SCD and depressive symptoms. Nevertheless, SCD-related cognitive alterations were present even when statistically controlling for depressive symptoms, highlighting the distinctiveness of the two concepts. In the AD field, SCD was found to precede the development of depressive symptoms^33^. Unfortunately, our sample characterization lacks a dedicated quantitative anxiety assessment. This represents a limitation, as anxiety - alongside depressive symptoms - is one of the major neuropsychiatric factors associated with SCD^11^ and should ideally be controlled for when investigating objective correlates of SCD. In the absence of objective impairment, anxiety-related worry may contribute to the endorsement of SCD, introducing a potential circularity in SCD classifications. Future studies should include validated scales to differentiate the influence of specific affective symptoms (e.g., anxiety, depression) on SCD.

Interestingly, we did not observe significant differences in cognitive performance between HC and either RBD.SCD– or RBD.SCD+ groups. This may reflect the relatively mild nature of very early cognitive symptoms and thus the small effect sizes observed across comparisons, or limitations in group characterization. Specifically, the HC group was not screened using biomarker evidence or polysomnography, which may have resulted in the inclusion of individuals with undetected sleep disorders or early neurodegenerative changes not captured by medical history and cognitive assessments. While meta-analytic evidence suggests that individuals with iRBD perform worse than HC across multiple cognitive domains^4^, our findings call this assumption into question. A possible explanation is the frequent neglect of cognitive heterogeneity (i.e., age-adequate cognition, SCD, and MCI) within iRBD samples in prior research.

In VBM (voxel-wise and ROI-analysis) only one suprathreshold cluster following corrections for multiple comparisons was identified. However, the clusters identified in the parametric voxel-wise and ROI-analyses as well as the non-parametric uncorrected results further align with the literature^16,17^. Our results particularly revealed reduced GM volume in RBD.SCD+ compared to RBD.SCD– for the left SFG. This region, including the dorsolateral prefrontal cortex, was identified as a brain structural correlate of SCD before^16,17^. Generally, it is associated with higher cognitive functions, including attention, working memory, cognitive control^34^, and interoception^35^. Notably, a general convergence of cognitive and affective functions in the left SFG is discussed^36^. Recently, two brain atrophy progression subtypes underlying phenoconversion in iRBD have been proposed, the “cortical-first” progression subtype and a “subcortical-first” progression subtype^37^. In the cortical-first subtype, which is linked to an increased likelihood of developing DLB, atrophy begins in the frontal lobe, spreading to temporal and parietal regions before affecting subcortical structures^37^. The present findings may tentatively suggest that SCD could be an early marker of the cortical-first subtype, given the partial overlap in brain signatures. However, this hypothesis requires further investigation in larger, longitudinal samples.

Longitudinally, the presence of SCD in cognitively healthy individuals with manifest PD increased the risk of progressing to MCI or dementia within ~3 years by factor 2.71 [95%CI:1.82;4.04]^11^. These findings align with evidence from the AD field^14,18^. Future longitudinal studies on SCD in iRBD are warranted to evaluate the prognostic potential of SCD to predict the development of MCI and phenoconversion to advanced α-synucleinopathies. The investigation of determinants of SCD in iRBD and the potential role of these determinants in moderating the impact of SCD as an early marker and potential precursor of MCI will be of high interest for developing targets for (secondary) prevention and identifying individuals at high risk for clinical progression.

The strengths of this study include the thorough assessment of objective and subjective cognitive functioning in our sample, paired with standardized clinical assessments and optional 3T structural MRI. However, since the primary study^24^ was not specifically designed to assess SCD, the Multi-SubCoDE was the only available quantitative measure of SCD. This limits the ability to assess convergent validity with other established instruments in the current iRBD sample. Further, the sample size limits the conclusions that can be drawn, especially considering the rather subtle alterations associated with the presence of SCD. In particular, the investigation of neurobiological correlates of SCD needs larger samples. Furthermore, our results may be biased towards overestimating the prevalence and the objective correlates of SCD: The present sample consists of volunteers of the local iRBD cohort participating in a clinical trial on cognitive training and promoting a healthy lifestyle^24^. The nature of this trial may have attracted people who already experience subtle cognitive decline or are concerned about (possible future) cognitive decline. Notably, individuals in our iRBD group were not newly diagnosed cases. Despite the commonly observed diagnostic delay in RBD^38^ and considering phenoconversion rates following the iRBD diagnosis^39^, our sample may still be biased towards more benign iRBD subtypes^21^. Future replications of these findings in population-based samples and multi-centric projects on SCD as an early marker of objective cognitive impairment in iRBD are warranted. Future studies should also include HC groups characterized by biomarker evidence and comprehensive sleep evaluation using polysomnography, whose absence represents an additional limitation of the present study.

To conclude, SCD may be an early marker of objectively quantifiable MCI in individuals with iRBD, as it was associated with subtle changes in cognitive functioning. Furthermore, the presence of SCD in iRBD was associated with more pronounced non-motor symptoms such as depressive symptoms, and may reflect subtle brain structural changes. SCD should be included as a standard assessment in cohort studies focusing on early α-synucleinopathies to increase our understanding of the role of SCD as a prodromal marker of MCI and conversion to advanced α-synucleinopathies.

## Methods

### Study design and participants

This cross-sectional study utilized data assessed between 06/2022 and 12/2024 during the baseline assessment of the CogTrAiL-RBD randomized controlled trial at the University Hospital Cologne in Germany^24^. Participants were recruited from our local iRBD cohort^40^ and HC via newsletters and flyers. Reporting of this study follows the STrengthening the Reporting of OBservational studies in Epidemiology (STROBE) guidelines (Supplementary Table 5)^41^.

Individuals with iRBD fulfilled the following inclusion criteria: (i) diagnosis of iRBD confirmed by video-polysomnography, (ii) age between 40 and 80 years, (iii) normal or corrected-to-normal vision and hearing, and (iv) German as native tongue or sufficient proficiency in German. Exclusion criteria for individuals with iRBD were: (i) severe cognitive dysfunctions (Montréal Cognitive Assessment, MoCA, ≤22)^42^ interfering with the ability to give informed consent, (ii) significant neurological and psychiatric concomitant diseases (including any motor syndrome meeting diagnostic criteria, e.g., for PD), and for those willing to participate in the optional MRI module, (iii) contraindications for MRI. The same inclusion and exclusion criteria were applied for HC plus the absence of diagnoses of movement disorders, signs of iRBD, or any other psychiatric and neurological condition (including MCI as assessed by Level-II cognitive assessment).

For the present analyses, we used all available cases with completed neuropsychological testing and available questionnaire data, leading to an exclusion of two individuals with iRBD, who did not comply to fill out the questionnaires. This resulted in a final sample size of *N* = 80 individuals with iRBD and *N* = 27 HC.

### Standard protocol approvals, registrations, and patient consents

Ethical approval was granted by the ethics committee of the Medical Faculty of the University of Cologne on 2022–03-09 (Identifier: 21–1291). The study was conducted in accordance with the principles outlined in the Declaration of Helsinki. All participants gave written informed consent. The clinical trial^24^ of which baseline data was analyzed for the present study, was prospectively registered in the German Clinical Trial Register on 2022–03-11 (DRKS00024898, https://drks.de/search/de/trial/DRKS00024898).

### Assessments

All subjects participated in clinical and neuropsychological assessments and, if willing, an optional MRI module. Following the in-person assessments, participants digitally filled out questionnaires on various non-motor symptoms (for a complete list of assessments, see Supplementary Table 6).

#### Clinical and motor assessments

Depressive symptoms were assessed with the BDI-II^43^ and the self-reported presence of non-motor symptoms with the Movement Disorder Society (MDS) NMSQ^44^. In addition to reporting the total NMSQ score, we also present response distributions to individual items addressing memory problems, attentional deficits, and anxiety. PD-related motor impairment was assessed with the MDS-UPDRS-III^45^. The Purdue Pegboard (dominant hand) was utilized to assess fine motor abilities^46^.

#### Cognitive assessments

The MoCA^42^ was applied as a cognitive screening. According to the MDS guidelines for the operationalization of MCI in PD^12^, the Level-II cognitive battery included at least two tests for each of the five main cognitive domains (executive functions, attention & working memory, memory, visuo-cognition, language) and one additional test for social cognition. Test scores were demographically adjusted and standardized using published normative data and transformed into *z*-scores during data preprocessing. An overview of the assignment of cognitive tests to the respective domains is presented in Supplementary Table 5. Equally-weighted cognitive domain composite scores were calculated as the mean *z*-score of tests within one cognitive domain. Furthermore, an equally-weighted global cognition composite score was calculated based on the cognitive domain composite scores.

#### Assessment of subjective cognitive decline

The Multi-SubCoDE was utilized to assess multi-domain SCD. Prior versions of the Multi-SubCoDE were previously used and validated in local research studies^21–23^. The Multi-SubCoDE is aligned with the SCD framework introduced by the SCD-I^7^ and adopts the interview format of the Subjective Cognitive Decline Interview^47^ introduced in the DELCODE study^48^. The translated English version of the Multi-SubCoDE is presented in Table 2 (for the original German version, see Supplementary Table 7 and Supplementary Table 8).

**Table 2:** English version of the Multi-Domain Subjective Cognitive Decline Evaluation (Multi-SubCoDE)

Domain	General question	Specific questions		
Memory	A. Do you feel like your memory is deteriorating?^b^			
		A1. Have you found it harder recently to remember events that occurred a short while ago? A2. Have you found it harder recently to remember where you have placed certain items? A3. Have you found it harder recently to remember the contents of a conversation after a few days?		
Attention / Processing speed	B. Have you noticed a decline in your attention span recently?^b^			
		B1. Have you needed more time recently to complete tasks? B2. Have you made more mistakes recently when completing tasks? B3. Have you found it difficult recently to finish a task without getting distracted?		
Language^a^	C. Have you observed any language difficulties recently, such as trouble forming sentences or understanding spoken language?^b^			
		C1. Have you experienced difficulty recently in recalling specific words during conversations? C2. Have you struggled recently to recall the name of an object even when it is in front of you? C3. Have you found it difficult recently to understand what someone is trying to communicate or explain to you?		
Executive functions	D. Have you noticed increased difficulty recently in managing complex everyday tasks?^b^			
		D1. Have you found it harder recently to pay attention to multiple things at once? D2. Have you had more difficulty recently in thinking ahead and making prudent decisions? D3. Have you found it more challenging recently to engage with new activities or topics?		
Visuo-cognition	E. Have you observed a decline recently in your spatial thinking and visualization abilities?^b^			
		E1. Have you had more trouble recently orienting yourself in your surroundings? E2. Have you had more trouble recently understanding information from maps, plans, or graphical representations? E3. Have you had more trouble recently estimating distances, sizes, quantities, distances, or proportions accurately?		
Social cognition	F. Do you feel that your personality and/or behavior toward others has changed recently?^b^			
		F1. Have you found it difficult recently to interpret what someone is trying to express through facial expressions and gestures? F2. Have you found it difficult recently to interpret others’ behavior and respond appropriately? F3. Have you noticed increased difficulty recently in empathizing with and understanding the moods and feelings of other people?		

	Response options	Number of questions	Subscore	Max. score
*General questions* (A–F)	[0] No. [1] Yes.	6	**SCD-Domains**	**6**
*For each General Question (A–F)* If yes…				
Does this change worry you?	[0] No. [1] Yes, mildly. [2] Yes, severely.	max. 6	**SCD-Worries**	**12**
When did you start noticing this change?	[0] In the last three months. [1] In the last year. [2] For more than a year.	max. 6	**SCD-Time**	**12**
Has someone close to you ever pointed out this change to you?	[0] No. [1] Yes.	max. 6	**SCD-Confirmed**	**6**
*Specific questions* (A1–F3)		18 across domains:	**SCD-Severity**	**54**
A1–A3: Memory B1–B3: Attention / Processing Speed C1–C3: Language D1–D3: Executive Functions E1–E3: Visuo-Cognition F1–F3: Social Cognition	[0] No. [1] Sometimes. [2] Often. [3] Always.	3 Memory 3 Attention / Processing Speed 3 Language 3 Executive Functions 3 Visuo-Cognition 3 Social Cognition	**SCD-Memory** **SCD-Attention / Processing Speed** **SCD-Language** **SCD-Executive Functions** **SCD-Visuo-Cognition** **SCD-Social Cognition**	**9** **9** **9** **9** **9** **9**

^a^The questions displayed in the table (C and C1) were now adapted – in the previous version, also used in the CogTrAiL-RBD study, the following questions were asked: C. Have you observed any difficulties in speaking recently?; C1. Have you experienced difficulty recently in recalling specific words during conversations?

^b^If yes, follow-up questions on (i) the presence of worries related to the perceived change (“Does this change worry you?”), (ii) the reference time frame (“When did you start noticing this change?”) and (iii) the confirmation by an informant (“Has someone close to you ever pointed out this change to you?”) are being asked.

Self-rated general SCD was assessed with six dichotomous questions (e.g., “Do you feel like your memory is deteriorating?”, 0–no, 1–yes) on six cognitive domains (memory, attention/processing speed, language, executive functions, visuo-cognition, and social cognition). The SCD-Domains score was computed as the sum of subjectively impaired cognitive domains (max. 6). For each subjectively impaired domain, follow-up questions on the presence of worries related to the perceived change (“Does this change worry you?”), the reference time frame (“When did you start noticing this change?”), and the confirmation by an informant (“Has someone close to you ever pointed out this change to you?”) were asked. For these follow-up questions, three scores were computed: SCD-Worries (max. 12), SCD-Time (max. 12), and SCD-Confirmed (max. 6).

In order to better illustrate difficulties in these general domains, three specific questions on self-rated SCD in each respective domain were asked (e.g., for the memory domain: “Have you found it harder recently to remember events that occurred a short while ago?”, “Have you found it harder recently to remember where you have placed certain items?”, “Have you found it harder recently to remember the contents of a conversation after a few days?”). The specific questions refer to examples from everyday life, as for example also used in the Everyday Cognition (ECog)^49^ questionnaire. The specific questions could be answered on a 4-point Likert-scale 0–no, 1–sometimes, 2–often, 3–always. There were 18 specific SCD-related questions, resulting in the SCD-Severity score with a maximum of 54. Furthermore, domain-specific scores were built, one for each assessed SCD-domain.

#### Magnetic resonance imaging

Brain imaging with a 3T SIEMENS PRISMA scanner equipped with a 64-channel head coil was conducted at the Research Center Juelich for all willing participants. To assess structural brain changes associated with SCD in iRBD, T1-weighted brain images of *n* = 35 individuals with iRBD without MCI were collected and acquired using a magnetization-prepared rapid acquisition gradient echo (MPRAGE) sequence (2500 ms repetition time, 2.22 ms echo time, 7° flip angle, 195.5 × 241 × 241 mm field of view, 208 × 256 × 256 matrix size, voxel size: 0.94 × 0.94 × 0.94 mm) within 4.58 ± 5.95 days of the clinical assessments. Images were preprocessed using the Computational Anatomy Toolbox (CAT12, CAT12.9 version 2560)^50^. Images were reoriented and aligned to the anterior commissure, followed by segmentation into GM, white matter (WM), and cerebrospinal fluid (CSF). The resulting GM images were normalized to the MNI space, modulated using the Jacobian determinant, and smoothed using a Gaussian kernel with a value of 6 mm full width at half maximum (FWHM). Additionally, projection-based cortical thickness was estimated, reconstructed, and smoothed with 15 mm FWHM.

### Classification of mild cognitive impairment and subjective cognitive decline

Individuals with iRBD were classified into three distinct categories: iRBD without SCD (RBD.SCD–), iRBD with SCD (RBD.SCD+), and iRBD with MCI (RBD.MCI). MCI was defined as the combination of (i) the presence of impaired test performance ≥1 Standard Deviation (SD, *z* ≤ –1) below published normative data in at least two tests within one or more of the five cognitive domains (Level-II, specific tests are indicated in Supplementary Table 1) and (ii) preserved functional independence^12^.

MCI was excluded before participants were classified as either RBD.SCD– or RBD.SCD+. SCD was determined based on the Multi-SubCoDE SCD-Domains score. A cut-off of 1.5 in the SCD-Domains score was previously used to classify SCD in individuals with PD^22^. In the current study, we aimed to validate - and if necessary, refine - this threshold for application in iRBD. Therefore, the optimal cut-off for the SCD-classification was evaluated by receiver operating characteristic (ROC-)analyses referencing two SCD-related questions from the NMSQ as an external comparator (for details, see Supplementary Material 9). Results supported retaining the 1.5 cut-off to classify someone as “without SCD” (SCD–, SCD-Domains ≤1) or “with SCD” (SCD+, SCD-Domains ≥2). However, a subjectively reported decline in only one cognitive domain was still acknowledged as SCD+ if this rating was accompanied by at least mild worries (SCD-Domains = 1 & SCD-Worries ≥1) to increase overall sensitivity and to adequately acknowledge the prognostic value of reported worries in the context of SCD^25^. To describe the pattern of SCD across domains, SCD profiles were defined using a framework similar to that described in MCI subtype classification^30,51^ based on mnestic involvement (amnestic “a” vs. non-amnestic “na” SCD) and the number of affected domains (single-domain “sd” vs. multi-domain “md” SCD), resulting in four SCD profiles: na-sd-SCD+, a-sd-SCD+, na-md-SCD+, a-md-SCD+.

### Statistical analyses

Data were analyzed using R (version 4.5.0)^52^ and CAT12 (CAT12.9 version 2560)^50^.

#### Characterization of subjective cognitive decline in iRBD

To characterize SCD in individuals with iRBD, the prevalence of SCD in general and SCD profiles based on involved domains as assessed in the Multi-SubCoDE are reported. The Multi-SubCoDE scores were compared between HC and individuals with iRBD without and with MCI using ANOVA models and post hoc *t*-tests. Partial eta squared (_p_η^2^) and Cohen’s *d* were reported as effect sizes accordingly, indicating small (_p_η^2^ ≥ 0.01, Cohen’s *d* ≥ 0.2), medium (_p_η^2^ ≥ 0.06, Cohen’s *d* ≥ 0.5) and large (_p_η^2^ ≥ 0.14, Cohen’s *d* ≥ 0.8) effects. The significance level was adjusted using the Benjamini–Hochberg procedure controlling the false discovery rate (FDR) across multiple comparisons.

#### Correlates of subjective cognitive decline in iRBD

Clinical characteristics and cognitive performance were compared between HC, RBD.SCD–, RBD.SCD+, and RBD.MCI with ANOVA models as described above. Cognitive performance was compared between groups with ANCOVA models adjusted for depressive symptoms operationalized by the BDI-II total score. FDR-corrected post hoc *t*-tests based on the EMM from the models were performed. Additionally, a global MANCOVA model per group comparison was built across the cognitive outcomes (i.e., MoCA and six cognitive domain scores). Furthermore, ANCOVA models with age and the BDI-II total score as covariates were specified to investigate group differences between the RBD.SCD– and RBD.SCD+ group in GM volume (VBM) and cortical thickness (SBM). Besides, total intracranial volume, calculated using the CAT12, was included as a covariate in the VBM analysis. The resulting second-level models were analyzed using non-parametric permutation tests with 5000 permutations performed by the TFCE toolbox included in CAT12. Both, the parametric and non-parametric voxel-wise analyses are reported. The statistical significance threshold was set to *p* < 0.05 (FDR-corrected). Whole-brain ROI-analyses (i.e., without pre-selection of ROIs) based on the Neuromorphometrics atlas for volume data and the Desikan–Killiany atlas for surface data with statistical significance threshold set to *p* < 0.05 (Holm–Bonferroni-corrected) were performed to validate and further explore findings from the voxel-wise analyses.

## Supplementary information

Supplementary Information

## Data Availability

The data supporting this study’s findings are available on reasonable request from the corresponding author AO. The data are not publicly available due to privacy and ethical restrictions, as full anonymization is currently not feasible while the larger clinical trial remains ongoing.

## Code availability

The code supporting this study’s findings is available on reasonable request from the corresponding author AO.
