# Supplementary material for: Subjective cognitive decline in individuals with isolated REM sleep behavior disorder
Source: NPJ Parkinsons Dis. 2025 Oct 7;11:287. doi: 10.1038/s41531-025-01161-2 (PMC12504634; doi:10.1038/s41531-025-01161-2)
Supplement: Supplementary file 1 — Supplementary Information [file 41531_2025_1161_MOESM1_ESM.pdf]

SUPPLEMENTARY MATERIAL

Subjective cognitive decline in individuals with isolated REM sleep behavior disorder

Ophey et al.

Supplementary Table 1. Sample Characteristics of HC, RBD without MCI, and RBD.MCI

Supplementary Table 2. Test statistics for pairwise comparisons of the ANOVA models comparing HC, RBD without MCI, and RBD.MCI

Supplementary Table 3. Test statistics for pairwise comparisons of the ANOVA and ANCOVA models comparing HC, RBD.SCD–, RBD.SCD+, and RBD.MCI

Supplementary Table 4. Region-of-interest analyses for voxel- and surface-based morphometry

Supplementary Table 5. STrengthening the Reporting of OBservational studies in Epidemiology (STROBE) checklist

Supplementary Table 6. Neuropsychological and motor assessments in CogTrAiL-RBD

Supplementary Table 7. German Version of the Multi-Domain Subjective Cognitive Decline Evaluation (Multi-SubCoDE)

Supplementary Table 8. German Version of Response Options and the Scoring System of the Multi-Domain Subjective Cognitive Decline Evaluation (Multi-SubCoDE)

Supplementary Material 9. ROC-Analysis to determine a cut-off for the classification of Subjective Cognitive Decline assessed with the Multi-SubCoDE

Supplementary Table 1. Sample Characteristics of HC, RBD without MCI, and RBD.MCI

		**HC** *n* = 27	**RBD** *n* = 56	**RBD.MCI** *n* = 24	ANOVA Main Effect Group
Age *in years*		67.02 (4.86) [58.04 - 77.97]	69.75 (5.98) [55.90 - 80.93]	67.92 (5.72) [59.15 - 79.02]	$F(2,104) = 2.36$, $p = .1$, $_p\eta^2 = 0.04$
Sex, n (%)	female	3 (11.11%)	8 (14.29%)	2 (8.33%)	$\chi^2(2) = 0.59$, $p = .743$
	male	24 (88.89%)	48 (85.71%)	22 (91.67%)	
Education *in years*		17.06 (3.54) [11.50 - 27.00]	16.08 (2.92) [10.00 - 22.00]	14.71 (3.14) [7.00 - 21.00]	**$F(2,104) = 3.59$, $p = .031$, $_p\eta^2 = 0.06$**
Time since first reported RBD symptoms *in years*			9.93 (6.31) [1.92 - 30.93]	8.80 (6.44) [2.39 - 27.34]	$F(1,65) = 0.45$, $p = .506$, $_p\eta^2 = 0.01$
RBDSQ total score		1.77 (1.37) [0 - 5]	9.02 (2.87) [0 - 13]	8.26 (2.49) [2 - 12]	**$F(2,101) = 78.14$, $p < .001$, $_p\eta^2 = 0.61$**
MDS-UPDRS-III total score		4.00 (3.93) [0 - 14]	5.06 (4.63) [0 - 19]	5.09 (4.39) [1 - 17]	$F(2,63) = 0.4$, $p = .675$, $_p\eta^2 = 0.01$
Purdue Pegboard, dominant hand		-0.07 (0.85) [-1.93 - 1.38]	-0.71 (0.77) [-3.25 - 1.15]	-1.08 (1.02) [-3.03 - 0.70]	**$F(2,104) = 9.35$, $p < .001$, $_p\eta^2 = 0.15$**
BDI-II total score		1.41 (1.80) [0 - 7]	5.64 (5.65) [0 - 28]	7.00 (6.67) [0 - 25]	**$F(2,104) = 8.47$, $p < .001$, $_p\eta^2 = 0.14$**
NMSQ total score		2.15 (1.90) [0 - 8]	6.50 (3.54) [0 - 15]	7.04 (4.24) [0 - 18]	**$F(2,104) = 18.13$, $p < .001$, $_p\eta^2 = 0.26$**
NMSQ Memory Problems, Item 12		2 (7.4%)	21 (37.5%)	11 (45.8%)	**$\chi^2(2) = 10.160$, $p = .006$**
NMSQ Attentional Difficulties, Item 15		1 (3.7%)	14 (25.0%)	10 (41.7%)	**$\chi^2(2) = 9.827$, $p = .007$**
NMSQ Anxiety, Item 17		0 (0.0%)	3 (5.4%)	2 (8.3%)	$\chi^2(2) = 2.020$, $p = .364$
Multi-SubCoDE scores					
SCD-Domains		0.15 (0.36) [0 - 1]	1.27 (1.45) [0 - 5]	1.46 (1.32) [0 - 4]	**$F(2,104) = 9.38$, $p < .001$, $_p\eta^2 = 0.15$**
SCD-Worries		0.00 (0.00) [0 - 0]	1.23 (1.65) [0 - 7]	1.17 (1.46) [0 - 5]	**$F(2,104) = 7.76$, $p = .001$, $_p\eta^2 = 0.13$**
SCD-Confirmed		0.04 (0.19) [0 - 1]	0.61 (0.91) [0 - 3]	0.50 (0.83) [0 - 3]	**$F(2,104) = 5.04$, $p = .008$, $_p\eta^2 = 0.09$**
SCD-Severity		3.04 (2.17) [0 - 7]	6.52 (4.66) [0 - 22]	7.92 (6.70) [0 - 23]	**$F(2,104) = 7.53$, $p = .001$, $_p\eta^2 = 0.13$**
MoCA total score		27.26 (1.91) [24 - 30]	26.61 (2.02) [23 - 30]	25.25 (1.82) [23 - 29]	**$F(2,104) = 7.05$, $p = .001$, $_p\eta^2 = 0.12$**
Global Cognition		0.52 (0.21) [0.16 - 1.01]	0.50 (0.27) [-0.01 - 1.23]	0.08 (0.31) [-0.44 - 0.74]	**$F(2,104) = 23.65$, $p < .001$, $_p\eta^2 = 0.31$**
Executive Functions		0.49 (0.35) [-0.06 - 1.19]	0.52 (0.39) [-0.63 - 1.25]	0.04 (0.60) [-1.19 - 1.42]	**$F(2,104) = 10.67$, $p < .001$ $_p\eta^2 = 0.17$**
Attention & Working Memory		0.69 (0.54) [-0.25 - 1.75]	0.62 (0.51) [-0.53 - 1.99]	0.22 (0.60) [-0.60 - 1.49]	**$F(2,104) = 5.83$, $p = .004$, $_p\eta^2 = 0.1$**
Memory		0.49 (0.61) [-0.69 - 1.50]	0.49 (0.51) [-0.69 - 1.68]	-0.22 (0.54) [-1.21 - 0.65]	**$F(2,104) = 16.12$, $p < .001$, $_p\eta^2 = 0.24$**
Visuo-Cognition		0.66 (0.29) [-0.06 - 1.32]	0.61 (0.35) [-0.38 - 1.32]	0.35 (0.47) [-0.38 - 1.37]	**$F(2,104) = 5.56$, $p = .005$, $_p\eta^2 = 0.1$**
Language		0.27 (0.26) [-0.25 - 0.71]	0.23 (0.35) [-0.74 - 1.06]	0.03 (0.36) [-0.83 - 0.55]	**$F(2,104) = 3.76$, $p = .027$, $_p\eta^2 = 0.07$**
Social Cognition		-0.39 (1.17) [-2.33 - 2.33]	-0.59 (0.91) [-2.33 - 1.88]	-0.82 (0.87) [-2.33 - 0.92]	$F(2,104) = 1.26$, $p = .288$, $_p\eta^2 = 0.02$

Notes. Data are mean (standard deviation) [range] unless indicated otherwise. The main effect of group (HC, RBD without MCI, and RBD.MCI) of an ANOVA model is reported. Significant models appear in bold and highlighted in grey. If $p < .010$, significant follow-up *t*-tests controlling the false discovery rate (FDR) across multiple comparisons are indicated as follows: [a] HC vs. RBD, [b] HC vs. RBD.MCI, [c] RBD vs. RBD.MCI. Full test statistics for each pairwise comparison are reported in Supplementary Table S6. BDI-II, Beck Depression Inventory; HC, healthy controls; MCI, mild cognitive impairment; MDS-UPDRS-III, Movement Disorder Society Unified Parkinson's Disease Rating Scale; MoCA, Montréal Cognitive Assessment; Multi-SubCoDE, Multi-Domain Subjective Cognitive Decline Evaluation; NMSQ, Non-Motor Symptoms Questionnaire; RBD, rem sleep behavior disorder; SCD, subjective cognitive decline.

Supplementary Table 2. Test statistics for pairwise comparisons of the ANOVA models comparing HC, RBD without MCI, and RBD.MCI

	**HC - RBD**	**HC - RBD.MCI**	**RBD - RBD.MCI**
Age *in years*	$t(82) = -2.05$, $p_{FDR} = .129$, $d = -0.23$	$t(50) = -0.56$, $p_{FDR} = .575$, $d = -0.08$	$t(79) = 1.32$, $p_{FDR} = .284$, $d = 0.15$
Education *in years*	$t(82) = 1.33$, $p_{FDR} = .187$, $d = 0.15$	**$t(50) = 2.67$, $p_{FDR} = .026$, $d = 0.37$**	$t(79) = 1.8$, $p_{FDR} = .113$, $d = 0.2$
Time since first reported RBD symptoms *in years*	n.a.	n.a.	$t(79) = 0.67$, $p_{FDR} = .506$, $d = 0.07$
RBDSQ total score	**$t(82) = -12.21$, $p_{FDR} < .001$, $d = -1.34$**	**$t(50) = -9.09$, $p_{FDR} < .001$, $d = -1.27$**	$t(79) = 1.22$, $p_{FDR} = .224$, $d = 0.14$
MDS-UPDRS-III total score	$t(82) = -0.85$, $p_{FDR} = .773$, $d = -0.09$	$t(50) = -0.65$, $p_{FDR} = .773$, $d = -0.09$	$t(79) = -0.02$, $p_{FDR} = .981$, $d = 0$
Purdue Pegboard, dominant hand	**$t(82) = 3.2$, $p_{FDR} = .003$, $d = 0.35$**	**$t(50) = 4.21$, $p_{FDR} < .001$, $d = 0.59$**	**$t(79) = 1.77$, $p_{FDR} = .08$, $d = 0.2$**
BDI-II total score	**$t(82) = -3.45$, $p_{FDR} = .001$, $d = -0.38$**	**$t(50) = -3.8$, $p_{FDR} = .001$, $d = -0.53$**	$t(79) = -1.06$, $p_{FDR} = .291$, $d = -0.12$
NMSQ total score	**$t(82) = -5.48$, $p_{FDR} < .001$, $d = -0.6$**	**$t(50) = -5.15$, $p_{FDR} < .001$, $d = -0.72$**	$t(79) = -0.66$, $p_{FDR} = .514$, $d = -0.07$
Multi-SubCoDE scores			
SCD-Domains	**$t(82) = -3.87$, $p_{FDR} < .001$, $d = -0.43$**	**$t(50) = -3.78$, $p_{FDR} < .001$, $d = -0.53$**	$t(79) = -0.63$, $p_{FDR} = .528$, $d = -0.07$
SCD-Worries	**$t(82) = -3.8$, $p_{FDR} = .001$, $d = -0.42$**	**$t(50) = -3$, $p_{FDR} = .005$, $d = -0.42$**	$t(79) = 0.19$, $p_{FDR} = .847$, $d = 0.02$
SCD-Confirmed	**$t(82) = -3.14$, $p_{FDR} = .007$, $d = -0.35$**	$t(50) = -2.13$, $p_{FDR} = .053$, $d = -0.3$	$t(79) = 0.57$, $p_{FDR} = .572$, $d = 0.06$
SCD-Severity	**$t(82) = -3.13$, $p_{FDR} = .003$, $d = -0.34$**	**$t(50) = -3.66$, $p_{FDR} = .001$, $d = -0.51$**	$t(79) = -1.21$, $p_{FDR} = .23$, $d = -0.13$
MoCA total score	$t(82) = 1.43$, $p_{FDR} = .156$, $d = 0.16$	**$t(50) = 3.67$, $p_{FDR} = .001$, $d = 0.51$**	**$t(79) = 2.85$, $p_{FDR} = .008$, $d = 0.32$**
Global Cognition	$t(82) = 0.23$, $p_{FDR} = .818$, $d = 0.03$	**$t(50) = 5.81$, $p_{FDR} < .001$, $d = 0.81$**	**$t(79) = 6.46$, $p_{FDR} < .001$, $d = 0.72$**
Executive Functions	$t(82) = -0.3$, $p_{FDR} = .762$, $d = -0.03$	**$t(50) = 3.64$, $p_{FDR} = .001$, $d = 0.51$**	**$t(79) = 4.47$, $p_{FDR} < .001$, $d = 0.5$**
Attention & Working Memory	$t(82) = 0.57$, $p_{FDR} = .573$, $d = 0.06$	**$t(50) = 3.1$, $p_{FDR} = .005$, $d = 0.43$**	**$t(79) = 3.02$, $p_{FDR} = .005$, $d = 0.34$**
Memory	$t(82) = -0.02$, $p_{FDR} = .985$, $d = 0$	**$t(50) = 4.68$, $p_{FDR} < .001$, $d = 0.66$**	**$t(79) = 5.4$, $p_{FDR} < .001$, $d = 0.6$**
Visuo-Cognition	$t(82) = 0.52$, $p_{FDR} = .606$, $d = 0.06$	**$t(50) = 3.01$, $p_{FDR} = .006$, $d = 0.42$**	**$t(79) = 2.97$, $p_{FDR} = .006$, $d = 0.33$**
Language	$t(82) = 0.5$, $p_{FDR} = .621$, $d = 0.05$	**$t(50) = 2.51$, $p_{FDR} = .027$, $d = 0.35$**	**$t(79) = 2.41$, $p_{FDR} = .027$, $d = 0.27$**
Social Cognition	$t(82) = 0.86$, $p_{FDR} = .394$, $d = 0.09$	$t(50) = 1.59$, $p_{FDR} = .346$, $d = 0.22$	$t(79) = 1$, $p_{FDR} = .394$, $d = 0.11$

Notes. Follow-up *t*-tests for the ANOVA models controlling the false discovery rate (FDR) across multiple comparisons are reported. Significant comparisons appear in bold and highlighted in grey. BDI-II, Beck Depression Inventory; HC, healthy controls; MCI, mild cognitive impairment; MDS-UPDRS-III, Movement Disorder Society Unified Parkinson's Disease Rating Scale; MoCA, Montréal Cognitive Assessment; Multi-SubCoDE, Multi-Domain Subjective Cognitive Decline Evaluation; NMSQ, Non-Motor Symptoms Questionnaire; RBD, REM sleep behavior disorder; RBDSQ, REM Sleep Behavior Disorder Screening Questionnaire; SCD, subjective cognitive decline.

Supplementary Table 3. Test statistics for pairwise comparisons of the ANOVA and ANCOVA models comparing HC, RBD.SCD–, RBD.SCD+, and RBD.MCI

	**HC - RBD.SCD–**	**HC - RBD.SCD+**	**HC - RBD.MCI**	**RBD.SCD– - RBD.SCD+**	**RBD.SCD– - RBD.MCI**	**RBD.SCD+ - RBD.MCI**
Age *in years*	$t(53) = -2.54$, $p_{FDR} = .076$, $d = -0.35$	$t(55) = -1.08$, $p_{FDR} = .422$, $d = -0.14$	$t(50) = -0.57$, $p_{FDR} = .636$, $d = -0.08$	$t(55) = 1.5$, $p_{FDR} = .273$, $d = 0.2$	$t(50) = 1.9$, $p_{FDR} = .182$, $d = 0.27$	$t(52) = 0.47$, $p_{FDR} = .636$, $d = 0.07$
Education *in years*	$t(53) = 0.31$, $p_{FDR} = .76$, $d = 0.04$	$t(55) = 1.98$, $p_{FDR} = .101$, $d = 0.26$	**$t(50) = 2.69$, $p_{FDR} = .049$, $d = 0.38$**	$t(55) = 1.67$, $p_{FDR} = .148$, $d = 0.22$	$t(50) = 2.4$, $p_{FDR} = .055$, $d = 0.34$	$t(52) = 0.82$, $p_{FDR} = .495$, $d = 0.11$
Time since first reported RBD symptoms *in years*	n.a.	n.a.	n.a.	$t(55) = 0.39$, $p_{FDR} = .697$, $d = 0.05$	$t(50) = 0.77$, $p_{FDR} = .697$, $d = 0.11$	$t(52) = 0.41$, $p_{FDR} = .697$, $d = 0.06$
RBDSQ total score	**$t(53) = -10.2$, $p_{FDR} < .001$, $d = -1.39$**	**$t(55) = -10.99$, $p_{FDR} < .001$, $d = -1.47$**	**$t(50) = -9.07$, $p_{FDR} < .001$, $d = -1.27$**	$t(55) = -0.7$, $p_{FDR} = .485$, $d = -0.09$	$t(50) = 0.73$, $p_{FDR} = .485$, $d = 0.1$	$t(52) = 1.41$, $p_{FDR} = .244$, $d = 0.19$
MDS-UPDRS-III total score	$t(53) = -0.62$, $p_{FDR} = .972$, $d = -0.08$	$t(55) = -0.81$, $p_{FDR} = .972$, $d = -0.11$	$t(50) = -0.65$, $p_{FDR} = .972$, $d = -0.09$	$t(55) = -0.14$, $p_{FDR} = .972$, $d = -0.02$	$t(50) = -0.09$, $p_{FDR} = .972$, $d = -0.01$	$t(52) = 0.04$, $p_{FDR} = .972$, $d = 0$
Purdue Pegboard, dominant hand	**$t(53) = 2.88$, $p_{FDR} = .015$, $d = 0.39$**	**$t(55) = 2.66$, $p_{FDR} = .018$, $d = 0.35$**	**$t(50) = 4.19$, $p_{FDR} < .001$, $d = 0.59$**	$t(55) = -0.27$, $p_{FDR} = .786$, $d = -0.04$	$t(50) = 1.4$, $p_{FDR} = .198$, $d = 0.2$	$t(52) = 1.68$, $p_{FDR} = .143$, $d = 0.23$
BDI-II total score	$t(53) = -0.85$, $p_{FDR} = .395$, $d = -0.12$	**$t(55) = -5.59$, $p_{FDR} < .001$, $d = -0.75$**	**$t(50) = -4.17$, $p_{FDR} < .001$, $d = -0.58$**	**$t(55) = -4.72$, $p_{FDR} < .001$, $d = -0.63$**	**$t(50) = -3.34$, $p_{FDR} = .002$, $d = -0.47$**	$t(52) = 1.18$, $p_{FDR} = .29$, $d = 0.16$
NMSQ total score	**$t(53) = -3.51$, $p_{FDR} = .001$, $d = -0.48$**	**$t(55) = -6.21$, $p_{FDR} < .001$, $d = -0.83$**	**$t(50) = -5.29$, $p_{FDR} < .001$, $d = -0.74$**	**$t(55) = -2.64$, $p_{FDR} = .014$, $d = -0.35$**	**$t(50) = -1.89$, $p_{FDR} = .074$, $d = -0.26$**	$t(52) = 0.64$, $p_{FDR} = .526$, $d = 0.09$
MANCOVA RESULTS: per group comparison across cognitive scores	$V = 0.13$, $F(8,44) = 0.82$, $p = .590$, $_p\eta^2 = 0.13$	$V = 0.093$, $F(8,45) = 0.58$, $p = .789$, $_p\eta^2 = 0.09$	**$V = 0.466$, $F(8,43) = 5.37$, $p < .001$, $_p\eta^2 = 0.47$**	$V = 0.192$, $F(8,45) = 1.34$, $p = .250$, $_p\eta^2 = 0.19$	**$V = 0.53$, $F(8,42) = 5.93$, $p < .001$, $_p\eta^2 = 0.53$**	**$V = 0.353$, $F(8,43) = 2.93$, $p = 0.010$, $_p\eta^2 = 0.35$**
MoCA total score*	$t(53) = 1.46$, $p_{FDR} = .219$, $d = 0.2$	$t(55) = 0.51$, $p_{FDR} = .614$, $d = 0.07$	**$t(50) = 3.12$, $p_{FDR} = .014$, $d = 0.44$**	$t(55) = -0.83$, $p_{FDR} = .488$, $d = -0.11$	$t(50) = 1.85$, $p_{FDR} = .135$, $d = 0.26$	**$t(52) = 2.85$, $p_{FDR} = .016$, $d = 0.39$**
Global Cognition*	$t(53) = -0.93$, $p_{FDR} = .355$, $d = -0.13$	$t(55) = 1.42$, $p_{FDR} = .189$, $d = 0.19$	**$t(50) = 5.68$, $p_{FDR} < .001$, $d = 0.79$**	**$t(55) = 2.34$, $p_{FDR} = .032$, $d = 0.31$**	**$t(50) = 6.69$, $p_{FDR} < .001$, $d = 0.94$**	**$t(52) = 4.63$, $p_{FDR} < .001$, $d = 0.64$**
Executive Functions*	$t(53) = -0.86$, $p_{FDR} = .467$, $d = -0.12$	$t(55) = 0.29$, $p_{FDR} = .774$, $d = 0.04$	**$t(50) = 3.37$, $p_{FDR} = .002$, $d = 0.47$**	$t(55) = 1.1$, $p_{FDR} = .412$, $d = 0.15$	**$t(50) = 4.26$, $p_{FDR} < .001$, $d = 0.6$**	**$t(52) = 3.36$, $p_{FDR} = .002$, $d = 0.46$**
Attention & Working Memory*	$t(53) = -0.01$, $p_{FDR} = .995$, $d = 0$	$t(55) = 1.8$, $p_{FDR} = .091$, $d = 0.24$	**$t(50) = 3.55$, $p_{FDR} = .002$, $d = 0.5$**	$t(55) = 1.86$, $p_{FDR} = .091$, $d = 0.25$	**$t(50) = 3.65$, $p_{FDR} = .002$, $d = 0.51$**	$t(52) = 1.9$, $p_{FDR} = .091$, $d = 0.26$
Memory*	$t(53) = -0.61$, $p_{FDR} = .714$, $d = -0.08$	$t(55) = -0.03$, $p_{FDR} = .973$, $d = 0$	**$t(50) = 3.97$, $p_{FDR} < .001$, $d = 0.56$**	$t(55) = 0.53$, $p_{FDR} = .714$, $d = 0.07$	**$t(50) = 4.65$, $p_{FDR} < .001$, $d = 0.65$**	**$t(52) = 4.37$, $p_{FDR} < .001$, $d = 0.6$**
Visuo-Cognition*	$t(53) = -0.04$, $p_{FDR} = .966$, $d = -0.01$	$t(55) = 1.51$, $p_{FDR} = .161$, $d = 0.2$	**$t(50) = 3.29$, $p_{FDR} = .004$, $d = 0.46$**	$t(55) = 1.6$, $p_{FDR} = .161$, $d = 0.21$	**$t(50) = 3.41$, $p_{FDR} = .004$, $d = 0.48$**	$t(52) = 1.93$, $p_{FDR} = .114$, $d = 0.26$
Language*	$t(53) = -0.56$, $p_{FDR} = .577$, $d = -0.08$	$t(55) = 1.23$, $p_{FDR} = .271$, $d = 0.16$	$t(50) = 2.35$, $p_{FDR} = .061$, $d = 0.33$	$t(55) = 1.79$, $p_{FDR} = .153$, $d = 0.24$	**$t(50) = 2.93$, $p_{FDR} = .025$, $d = 0.41$**	$t(52) = 1.22$, $p_{FDR} = .271$, $d = 0.17$
Social Cognition*	$t(53) = 0.11$, $p_{FDR} = .913$, $d = 0.01$	$t(55) = 0.48$, $p_{FDR} = .829$, $d = 0.06$	$t(50) = 0.99$, $p_{FDR} = .829$, $d = 0.14$	$t(55) = 0.4$, $p_{FDR} = .829$, $d = 0.05$	$t(50) = 0.92$, $p_{FDR} = .829$, $d = 0.13$	$t(52) = 0.55$, $p_{FDR} = .829$, $d = 0.08$

Notes. Test statistics of follow-up *t*-tests for the ANOVA or *ANCOVA models adjusted for depressive symptoms based on the estimated marginal means controlling the false discovery rate (FDR) across multiple comparisons unless indicated otherwise. Significant comparisons appear in bold and highlighted in grey. BDI-II, Beck Depression Inventory; HC, healthy controls; MCI, mild cognitive impairment; MDS-UPDRS-III, Movement Disorder Society Unified Parkinson's Disease Rating Scale; MoCA, Montréal Cognitive Assessment; NMSQ, Non-Motor Symptoms Questionnaire; RBD, REM sleep behavior disorder; RBDSQ, REM Sleep Behavior Disorder Screening Questionnaire; SCD, subjective cognitive decline.

Supplementary Table 4. Region-of-interest analyses for voxel- and surface-based morphometry comparing RBD.SCD– and RBD.SCD+

ROI-analysis based on Voxel-Based Morphometry

left hemisphere (P<0.05, uncorrected):					right hemisphere (P<0.05, uncorrected):				
T-value	Ze-value	P-value uncorrected	P-value Holm-Bonferroni corrected	Neuromorphometrics atlas	T-value	Ze-value	P-value uncorrected	P-value Holm-Bonferroni corrected	Neuromorphometrics atlas
**3.20**	**2.94**	**.0017**	**.0383**	**SFG superior frontal gyrus**	3.01	2.78	.0027	.1839	Amygdala
2.70	2.53	.0057	.4245	ITG inferior temporal gyrus	2.73	2.55	.0053	.2029	LOrG lateral orbital gyrus
2.36	2.24	.0127	.7479	LiG lingual gyrus	2.67	2.51	.0061	.1826	ITG inferior temporal gyrus
2.07	1.98	.0236	.7320	MSFG superior frontal gyrus medial segment	2.38	2.26	.0120	>.9999	Ventral DC
2.03	1.94	.0260	.4937	FuG fusiform gyrus	2.38	2.26	.0120	.5518	Ent entorhinal area
1.82	1.76	.0394	.5122	Ventral DC	2.31	2.20	.0140	>.9999	LiG lingual gyrus
1.80	1.74	.0409	>.9999	SMG supramarginal gyrus	2.29	2.17	.0149	.9072	MTG middle temporal gyrus
1.76	1.70	.0448	.9409	PoG postcentral gyrus	2.24	2.14	.0163	>.9999	TMP temporal pole
1.71	1.65	.0493	.7883	OpIFG opercular part of the inferior frontal gyrus	1.96	1.89	.0296	.5332	AnG angular gyrus
					1.95	1.88	.0302	>.9999	MOrG medial orbital gyrus
					1.95	1.88	.0303	.0607	SPL superior parietal lobule
					1.75	1.69	.0454	>.9999	Hippocampus
					1.74	1.68	.0460	>.9999	PHG parahippocampal gyrus

ROI-analysis based on Surface-Based Morphometry

left hemisphere (P<0.05, uncorrected):					right hemisphere (P<0.05, uncorrected):				
T-value	Ze-value	P-value uncorrected	P-value Holm-Bonferroni corrected	Desikan-Killiany atlas	T-value	Ze-value	P-value uncorrected	P-value Holm-Bonferroni corrected	Desikan-Killiany atlas
2.15	2.06	.0196	>.9999	lingual	2.32	2.21	.0136	.1900	medialorbitofrontal
2.03	1.95	.0257	>.9999	medialorbitofrontal	2.17	2.08	.0187	.5808	lateralorbitofrontal
					2.08	2.00	.0229	.3757	rostralanteriorcingulate

Supplementary Table 5. STrengthening the Reporting of OBservational studies in Epidemiology (STROBE) checklist

	Item No.	Recommendation	Page No.
**Title and abstract**	1	(*a*) Indicate the study's design with a commonly used term in the title or the abstract	2
		(*b*) Provide in the abstract an informative and balanced summary of what was done and what was found	2
**Introduction**			
Background/rationale	2	Explain the scientific background and rationale for the investigation being reported	3-5
Objectives	3	State specific objectives, including any prespecified hypotheses	4-5
**Methods**			
Study design	4	Present key elements of study design early in the paper	4-5, 13-14
Setting	5	Describe the setting, locations, and relevant dates, including periods of recruitment, exposure, follow-up, and data collection	13
Participants	6	(*a*) *Cohort study*—Give the eligibility criteria, and the sources and methods of selection of participants. Describe methods of follow-up<br>*Case-control study*—Give the eligibility criteria, and the sources and methods of case ascertainment and control selection. Give the rationale for the choice of cases and controls<br>*Cross-sectional study*—Give the eligibility criteria, and the sources and methods of selection of participants	13-14
		(*b*) *Cohort study*—For matched studies, give matching criteria and number of exposed and unexposed<br>*Case-control study*—For matched studies, give matching criteria and the number of controls per case	
Variables	7	Clearly define all outcomes, exposures, predictors, potential confounders, and effect modifiers. Give diagnostic criteria, if applicable	14-17 + Supplementary
Data sources/ measurement	8*	For each variable of interest, give sources of data and details of methods of assessment (measurement). Describe comparability of assessment methods if there is more than one group	*14-17* + Supplementary
Bias	9	Describe any efforts to address potential sources of bias	13-19
Study size	10	Explain how the study size was arrived at	n.a.
Quantitative variables	11	Explain how quantitative variables were handled in the analyses. If applicable, describe which groupings were chosen and why	17-19
Statistical methods	12	(*a*) Describe all statistical methods, including those used to control for confounding	17-19
		(*b*) Describe any methods used to examine subgroups and interactions	17-19
		(*c*) Explain how missing data were addressed	17-19
		(*d*) *Cohort study*—If applicable, explain how loss to follow-up was addressed<br>*Case-control study*—If applicable, explain how matching of cases and controls was addressed<br>*Cross-sectional study*—If applicable, describe analytical methods taking account of sampling strategy	n.a.
		(*e*) Describe any sensitivity analyses	17-19

**Results**			
Participants	13*	(a) Report numbers of individuals at each stage of study—eg numbers potentially eligible, examined for eligibility, confirmed eligible, included in the study, completing follow-up, and analysed	5-8, Table 1, Supplementary
		(b) Give reasons for non-participation at each stage	n.a.
		(c) Consider use of a flow diagram	n.a.
Descriptive data	14*	(a) Give characteristics of study participants (eg demographic, clinical, social) and information on exposures and potential confounders	Table 1 + Supplementary
		(b) Indicate number of participants with missing data for each variable of interest	n.a.
		(c) *Cohort study*—Summarise follow-up time (eg, average and total amount)	n.a.
Outcome data	15*	*Cohort study*—Report numbers of outcome events or summary measures over time	*n.a.*
		Case-control study—Report numbers in each exposure category, or summary measures of exposure	*n.a.*
		Cross-sectional study—Report numbers of outcome events or summary measures	Table 1 + Supplementary
Main results	16	(*a*) Give unadjusted estimates and, if applicable, confounder-adjusted estimates and their precision (eg, 95% confidence interval). Make clear which confounders were adjusted for and why they were included	5-8, Table 1 + Supplementary
		(*b*) Report category boundaries when continuous variables were categorized	n.a.
		(*c*) If relevant, consider translating estimates of relative risk into absolute risk for a meaningful time period	n.a.
Other analyses	17	Report other analyses done—eg analyses of subgroups and interactions, and sensitivity analyses	5-8, Supplementary
**Discussion**			
Key results	18	Summarise key results with reference to study objectives	8-9
Limitations	19	Discuss limitations of the study, taking into account sources of potential bias or imprecision. Discuss both direction and magnitude of any potential bias	9-13
Interpretation	20	Give a cautious overall interpretation of results considering objectives, limitations, multiplicity of analyses, results from similar studies, and other relevant evidence	9-13
Generalisability	21	Discuss the generalisability (external validity) of the study results	9-13
**Other information**			
Funding	22	Give the source of funding and the role of the funders for the present study and, if applicable, for the original study on which the present article is based	19

Supplementary Table 6. Neuropsychological and motor assessments in CogTrAiL-RBD

DOMAIN	Abbreviation	Assessment	Relevant for Level-II MCI Assessment	Reference
**COGNITION**				
**Overall Cognitive State**				
Subjective Cognition	Multi-SubCoDE	Multi-SubCoDE		used e.g. in Ophey, et al. [1], Kalbe, et al. [2], Seger, et al. [3], first published in this article
Global Cognition	MoCA	Montreal Cognitive Assessment: Version B or A (total score)		Nasreddine, et al. [4]
**Social Cognition**	RMET	Reading the Mind in the Eyes Test (number of correct responses)		Baron-Cohen, et al. [5], Kynast, et al. [6]
**Executive**				
Semantic fluency	RWT sem	Regensburger Wortflüssigkeitstest: Food or Animals (number of correct words)	X*	Aschenbrenner, et al. [7]
Phonemic fluency	RWT phon	Regensburger Wortflüssigkeitstest: P- or S-words (number of correct words)	X*	Aschenbrenner, et al. [7]
Set-Shifting	TMTB/A	Trail Making Test (TMT): TMT-B time in seconds / TMT-A time in seconds	X	Reitan [8]; Aebi [9]
Inhibition	Stroop-I	Stroop Interference (time in seconds)		Bäumler and Stroop [10]
Logical Reasoning	LPS-4	Leistungsprüfsystem 50+: Subtest 4, Fluid Reasoning, Version A or B (number of correctly identified errors)		Sturm, et al. [11]
**Visuo-cognition**				
Construction	ROCFT-Cop	Rey Osterrieth Complex Figure Test (ROCFT): Figure Copy (total score 18-point scoring system)	X	Rey [12], Strauss, et al. [13]
Perception	LPS-11	Leistungsprüfsystem 50+: Subtest 11, Visual Perception, Version A or B (number of correctly identified pictures)	X*	Sturm, et al. [11]
Spatial Perception	BJLO	Benton Judgment of Line Orientation, Version V or H (number of correctly named pairs of orientation)		Benton, et al. [14], Benton [15]
	LPS-7	Leistungsprüfsystem 50+: Subtest 7, Spatial Rotation, Version A or B (number of correctly identified mirrored items)	X*	Sturm, et al. [11]
**Attention & Working Memory**				
Working Memory	DSback	Wechsler Adult Intelligence Scale (WAIS): Digit Span backwards (number of correctly recalled digit spans)	X	Wechsler [16]
	BTA	Brief Test of Attention (number of correct counts)		Schretlen [17]
Processing Speed	TMT-A	Trail Making Test A (time in seconds)	X	Reitan [8]; Aebi [9]
Attention	Stroop-W	Stroop Word Reading (time in seconds)		Bäumler and Stroop [10]
	Stroop-C	Stroop Color Naming (time in seconds)		Bäumler and Stroop [10]
**Memory**				
Verbal Memory	DSforw	Wechsler Adult Intelligence Scale (WAIS): Digit Span forwards (number of correctly recalled digit spans)		Wechsler [16]
	VLMT-Learn	Verbaler Lern- und Merkfähigkeitstest (VLMT): Wordlist Learning, Version C or A (number of correctly recalled words in the learning phase trial 1-5)	X*	Helmstaedter and Durwen [18]
	VLMT-Rec	Verbaler Lern- und Merkfähigkeitstest (VLMT): Wordlist Delayed Recall, Version C or A (number of correctly recalled words in the delayed recall condition)	X*	Helmstaedter and Durwen [18]
Visuo-spatial Memory	ROCFT-Rec	Rey Osterrieth Complex Figure Test (ROCFT): Figure Delayed Recall (total score 18-point scoring system)	X	Rey [12], Strauss, et al. [13]
**Language**				
Naming	ACL-Naming	Aphasia Check List, Subtest Naming (total score)	X	Kalbe, et al. [19]
Semantic and Abstraction	WAIS	Wechsler Adult Intelligence Scale (WAIS): Similarities (total score)	X	von Aster and Neubauer [20]
**NON-MOTOR**				
Depressive Symptoms	BDI-II	Beck Depression Inventory (BDI-II)		Beck, et al. [21]
Non-motor symptoms	NMSQ	Non-Motor Symptoms Questionnaire (NMSQ)		Chaudhuri, et al. [22]
**MOTOR**				
PD Motor Symptoms	MDS-UPDRS-III	Movement Disorder Society Unified Parkinson’s Disease Rating Scale Part III		Goetz, et al. [23]
Fine Motor Skills	PPT	Purdue Pegboard Test		Tiffin and Asher [24], Agnew, et al. [25]
**SLEEP**				
RBD Symptoms	RBDSQ	REM sleep behavior disorder screening questionnaire		Stiasny-Kolster, et al. [26]

Notes. Tests relevant for MCI diagnosis are indicated with X or X*. According to the Level-II diagnostic framework for MCI in Parkinson’s disease[27], MCI was defined as the combination of (i) the presence of impaired test performance ≥1 Standard Deviation (SD, $z \leq -1$) below published normative data in at least two tests within one or more of the five cognitive domains. X* denotes highly similar tests or strongly correlated scores from the same test paradigm (RWT sem & RWT phon; LPS-7 & LPS-11; VLMT-Learn & VLMT-Rec). Impairments in such paired tests are counted only once toward the ≥2-test criterion (I.e., impairments are equally treated between either one or both being impaired), while increasing sensitivity for detecting cognitive impairment.

Supplementary Table 7. German Version of the Multi-Domain Subjective Cognitive Decline Evaluation (Multi-SubCoDE)

Domäne	Allgemeine Frage	Spezifische Fragen
Gedächtnis	A. Haben Sie das Gefühl, Ihr Gedächtnis wird schlechter?*	
		A1. Ist es für Sie in letzter Zeit schwieriger geworden, sich an kurz zurückliegende Ereignisse zu erinnern?
		A2. Ist es für Sie in letzter Zeit schwieriger geworden, sich daran zu erinnern, wo Sie bestimmte Gegenstände aufbewahrt haben?
		A3. Ist es für Sie in letzter Zeit schwieriger geworden, sich nach einigen Tagen noch an den Inhalt eines Gespräches zu erinnern?
Aufmerksamkeit / Verarbeitungsgeschwindigkeit	B. Haben Sie beobachtet, dass Ihre Aufmerksamkeit in der letzten Zeit nachlässt?*	
		B1. Benötigen Sie in letzter Zeit für die Erledigung von Aufgaben mehr Zeit?
		B2. Unterlaufen Ihnen in letzter Zeit bei der Erledigung von Aufgaben vermehrt Fehler?
		B3. Fällt es Ihnen in letzter Zeit schwer, eine angefangene Tätigkeit zielgerichtet und ohne abzuschweifen zu Ende zu bringen?
Sprache⁺	C. Haben Sie beobachtet, dass Sie in letzter Zeit Sprachschwierigkeiten, z.B. beim Formulieren von Sätzen oder beim Sprachverständnis, haben?*	
		C1. Passiert es Ihnen in letzter Zeit häufiger, dass Ihnen im Gespräch mit anderen ein Wort nicht einfällt oder Sie Probleme haben Sätze zu bilden?
		C2. Passiert es in letzter Zeit häufiger, dass Sie einen Gegenstand vor Augen haben, Ihnen aber der Begriff dazu nicht einfällt?
		C3. Passiert es in letzter Zeit häufiger, dass Sie Probleme haben zu verstehen, was eine andere Person Ihnen mitteilen oder erklären möchte?
Exekutivfunktionen	D. Haben Sie beobachtet, dass Ihnen in letzter Zeit die Erledigung von komplexen Alltagsdingen vermehrt Probleme bereitet?*	
		D1. Haben Sie in letzter Zeit Probleme, wenn Sie bei einer Aufgabe verschiedene Dinge gleichzeitig beachten müssen?
		D2. Haben Sie in letzter Zeit vermehrt Probleme, vorausschauend und umsichtig zu denken und Entscheidungen zu treffen?
		D3. Haben Sie in letzter Zeit vermehrt Probleme, wenn Sie sich mit neuen Dingen oder Themen beschäftigen?
Visuo-Kognition	E. Haben Sie beobachtet, dass Ihr räumliches Denk- und Vorstellungsvermögen in letzter Zeit nachlässt?*	
		E1. Haben Sie in letzter Zeit Probleme, sich in Ihrer näheren Umgebung zu orientieren?
		E2. Haben Sie in letzter Zeit Probleme, die Information von Karten, Plänen oder grafischen Abbildungen zu erfassen?
		E3. Haben Sie in letzter Zeit Probleme, Entfernungen oder Größen, Mengen, Abstände, Proportionen richtig einzuschätzen?
Soziale Kognition	F. Haben Sie das Gefühl, dass sich Ihre Persönlichkeit und/oder Ihr Verhalten gegenüber anderen Personen in letzter Zeit verändert hat?*	
		F1. Haben Sie in letzter Zeit Probleme zu erkennen, was eine andere Person durch Gesichtsausdruck, Gestik oder Mimik zum Ausdruck bringen möchte?
		F2. Haben Sie in letzter Zeit Schwierigkeiten, das Verhalten von Personen richtig zu deuten und in angemessener Weise darauf zu reagieren?
		F3. Haben Sie in letzter Zeit beobachtet, dass es Ihnen schwer fällt, sich in die Stimmung und Gefühle anderer Personen hineinzudenken und diese zu deuten?

* wenn ja, werden Folgefragen (i) zur Präsenz von Sorgen bezogen auf die beobachtete Veränderung ("Bereitet Ihnen diese Veränderung Sorgen?"), (ii) dem Referenz-Zeitrahmen ("Seit wann ungefähr beobachten Sie diese Veränderung?") und (iii) der Bestätigung durch Angehörige ("Hat ein Angehöriger Sie schon einmal auf diese Veränderung hingewiesen?") gestellt. Details zum Scoring System sind in Supplementary Table 8 zu finden.

⁺ Frage C und C1 wurden ausgehend von früheren Versionen des Multi-SubCoDE überarbeitet: C. Haben Sie beobachtet, dass Sie in letzter Zeit beim Sprechen Schwierigkeiten haben?; C1. Passiert es Ihnen in letzter Zeit häufiger, dass Ihnen im Gespräch mit anderen ein Wort nicht einfällt?

Supplementary Table 8. German Version of Response Options and the Scoring System of the Multi-Domain Subjective Cognitive Decline Evaluation (Multi-SubCoDE)

	Antwortoptionen	Anzahl der Fragen	Subscore	Max. Score
**Allgemeine Fragen** (A-F)	[0] Nein.<br>[1] Ja.	6	**SCD-Domains**	**6**
Für jede der allgemeinen Fragen (A-F)<br>Wenn ja…				
Bereitet Ihnen diese Veränderung Sorgen?	[0] Nein.<br>[1] Ja, leichte Sorgen.<br>[2] Ja, schwere Sorgen.	max. 6	**SCD-Worries**	**12**
Seit wann ungefähr beobachten Sie diese Veränderung?	[0] In den letzten drei Monaten.<br>[1] Im letzten Jahr<br>[2] Seit mehr als einem Jahr	max. 6	**SCD-Time**	**12**
Hat ein Angehöriger Sie schon einmal auf diese Veränderung hingewiesen?	[0] Nein.<br>[1] Ja.	max. 6	**SCD-Confirmed**	**6**
**Spezifische Fragen** (A1-F3)		18 über alle Domänen hinweg:	**SCD-Severity**	**54**
A1-A3: Gedächtnis	[0] Nein.	3 Gedächtnis	**SCD-Memory**	**9**
B1-B3: Aufmerksamkeit / Verarbeitungsgeschwindigkeit	[1] Trifft manchmal zu.	3 Aufmerksamkeit / Verarbeitungsgeschwindigkeit	**SCD-Attention / Processing Speed**	**9**
C1-C3: Sprache	[2] Trifft überwiegend zu.	3 Sprache	**SCD-Language**	**9**
D1-D3: Exekutivfunktionen	[3] Trifft völlig zu.	3 Exekutivfunktionen	**SCD-Executive Functions**	**9**
E1-E3: Visuo-Kognition		3 Visuo-Kognition	**SCD-Visuo-Cognition**	**9**
F1-F3: Soziale Kognition		3 Soziale Kognition	**SCD-Social Cognition**	**9**

Supplementary Material 9

ROC-Analysis to determine a cut-off for the classification of Subjective Cognitive Decline assessed with the Multi-SubCoDE

Previously, a cut-off of 1.5 in the Multi-Domain Subjective Cognitive Decline Evaluation (Multi-SubCoDE) SCD-Domains score was used to classify SCD in Parkinson's disease (PD).[1] Within the present manuscript, we further aimed to validate and probably refine this cut-off for iRBD. Therefore, the optimal cut-off of the SCD-Domains score of the Multi-SubCoDE for the SCD classification was evaluated with receiver operating characteristic (ROC-)analyses referencing two SCD-related questions of the Non-Motor Symptom Questionnaire (NMSQ)[22] as an external comparator:

- NMSQ item 12: *problems remembering things that have happened recently or forgetting to do things*
- NMSQ item 15: *difficulty concentrating or staying focused*

Note, that the NMSQ is not a designated SCD screening instrument, but rather focuses on global non-motor symptoms. It has been previously used as a proxy for SCD in PD, though.[28] As the primary study[29] was not specifically designed with a focus on SCD, the NMSQ was the only available instrument next to the Multi-SubCoDE, which contains questions focusing on SCD. Items in the NMSQ are answered dichotomously (*yes / no*). ROC analysis was performed for each item separately and a joint classification of SCD based on the two items, i.e., classifying someone as SCD if one or both NMSQ items were answered with *yes*. All subjects without mild cognitive impairment (MCI), i.e., both healthy controls (HC) and individuals with REM sleep behavior disorder (iRBD), were included in the ROC analysis (*n* = 83). The best cutoff for the SCD-Domains score of the Multi-SubCoDE was chosen based on Youden's index, i.e., the threshold that maximizes the distance of the ROC curve to the diagonal line. The table reports sensitivity, specificity, negative and positive predictive values, Youden's index, and the overall area under the curve (AUC) for the optimal cutoff and adjacent cutoffs of the SCD-Domains score of the Multi-SubCoDE based on answers to the indicated NMSQ items.

	NMSQ Item 12			NMSQ Item 15			Combined NMSQ Items: 12 and/or 15		
Cutoff	0.5	1.5	2.5	0.5	1.5	2.5	0.5	1.5	2.5
Sensitivity	0.67	0.50	0.29	0.81	0.81	0.56	0.67	0.52	0.33
Specificity	0.66	0.86	0.92	0.66	0.90	0.96	0.68	0.89	0.95
Accuracy	0.66	0.76	0.73	0.69	0.88	0.88	0.67	0.77	0.75
True negatives	39	51	54	44	60	64	38	50	53
True positives	16	12	7	13	13	9	18	14	9
False negatives	8	12	17	3	3	7	9	13	18
False positives	20	8	5	23	7	3	18	6	3
NPV	0.83	0.81	0.76	0.94	0.95	0.90	0.81	0.79	0.75
PPV	0.44	0.60	0.58	0.36	0.65	0.75	0.50	0.70	0.75
Youden	1.33	1.36	1.21	1.47	1.71	1.52	1.35	1.41	1.28
AUC		0.70			0.84			0.72	

Notes. AUC, area under the curve; NMSQ, non-motor symptom questionnaire; NPV, negative predictive value; PPV, positive predictive value.

Based on the confirmed the presence of "problems remembering things that have happened recently or forgetting to do things" (NMSQ item 12) and/or "difficulty concentrating or staying focused" (NMSQ item 15), the results supported retaining the 1.5 cut-off of the SCD-Domains score to classify someone as without SCD (SCD–, SCD-Domains ≤ 1) or with SCD (SCD+, SCD-Domains ≥ 2). However, a subjectively reported decline in only one cognitive domain was still acknowledged as SCD+, if this rating was accompanied by at least mild worries (SCD-Domains = 1 & SCD-Worries ≥ 1), as the 0.5 cutoff increased sensitivity substantially and adequately acknowledges the prognostic value of reported worries in the context of SCD.[30]

References

1 Ophey, A. *et al.* Neural correlates and predictors of subjective cognitive decline in patients with Parkinson's disease. *Neurol. Sci.* **43**, 3153-3163 (2022).

2 Kalbe, E. *et al.* Computerized cognitive training in healthy older adults: Baseline cognitive level and subjective cognitive concerns predict training outcome. *Health* **10**, 20-55 (2018). https://doi.org:10.4236/health.2017.101003

3 Seger, A. *et al.* Clinical subtypes in patients with isolated REM sleep behaviour disorder. *npj Parkinson's Disease* **9**, 155 (2023). https://doi.org:10.1038/s41531-023-00598-7

4 Nasreddine, Z. S. *et al.* The Montreal Cognitive Assessment, MoCA: A brief screening tool for mild cognitive impairment. *Journal of the American Geriatrics Society* **53**, 695-699 (2005). https://doi.org:10.1111/j.1532-5415.2005.53221.x

5 Baron-Cohen, S., Jolliffe, T., Mortimore, C. & Robertson, M. Another advanced test of theory of mind: Evidence from very high functioning adults with autism or Asperger syndrome. *Journal of Child psychology and Psychiatry* **38**, 813-822 (1997).

6 Kynast, J. *et al.* Age-and sex-specific standard scores for the Reading the Mind in the Eyes Test. *Front. Aging Neurosci.* **12**, 607107 (2021).

7 Aschenbrenner, S., Tucha, O. & Lange, K. (Hogrefe, Göttingen, Germany, 2000).

8 Reitan, R. *Trail Making Test: Manual for administration and scoring.* . (Reitan Neuropsychology Laboratory, 1992).

9 Aebi, C. *Validierung der neuropsychologischen Testbatterie CERAD-NP: eine Multi-Center Studie*, University of Basel, (2002).

10 Bäumler, G. & Stroop, J. *Farbe-Wort-Interferenztest nach JR Stroop (FWIT)*. (Hogrefe, Verlag für Psychologie, 1985).

11 Sturm, W., Willmes, K. & Horn, W. *Leistungsprüfsystem für 50–90jährige. Handanweisung*. (Hogrefe, 1993).

12 Rey, A. L'examen psychologique dans les cas d'encéphalopathie traumatique.(Les problems.). *Archives de psychologie* (1941).

13 Strauss, E., Sherman, E. M. & Spreen, O. *A compendium of neuropsychological tests: Administration, norms, and commentary*. (American chemical society, 2006).

14 Benton, A., Hannay, H. J. & Varney, N. R. Visual perception of line direction in patients with unilateral brain disease. *Neurology* **25**, 907-907 (1975).

15 Benton, A. L. *Contributions to neuropsychological assessment: A clinical manual*. (Oxford University Press, USA, 1994).

16 Wechsler, D. *WMS-R: Wechsler memory scale-revised: Manual*. (Psychological Corporation, 1984).

17 Schretlen, D. *Brief test of attention*. (Psychological Assessment Resources, 1989).

18 Helmstaedter, C. & Durwen, H. VLMT: Verbaler Lern-und Merkfähigkeitstest: Ein praktikables und differenziertes Instrumentarium zur Prüfung der verbalen Gedächtnisleistungen. *Schweizer Archiv für Neurologie, Neurochirurgie und Psychiatrie* (1990).

19 Kalbe, E., Reinhold, N., Brand, M. & Kessler, J. *Aphasie-Check-Liste (ACL): Protokollheft, Testheft, Lösungsfolien, Vorlagen, Manual*. (ProLog, Therapie-und Lernmittel, 2002).

20 von Aster, M. & Neubauer, A. *Wechsler-intelligenztest für erwachsene: WIE; manual; übersetzung und adaptation der WAIS-III von David Wechsler*. (Pearson Assessment & Information, 2009).

21 Beck, A. T., Steer, R. A. & Brown, G. K. Beck depression inventory-II. *San Antonio* **78**, 490-498 (1996).

22 Chaudhuri, K. R. *et al.* International multicenter pilot study of the first comprehensive self-completed nonmotor symptoms questionnaire for Parkinson's disease: the NMSQuest study. *Mov Disord* **21**, 916-923 (2006). https://doi.org:10.1002/mds.20844

23 Goetz, C. G. *et al.* Movement Disorder Society-sponsored revision of the Unified Parkinson's Disease Rating Scale (MDS-UPDRS): scale presentation and clinimetric testing results. *Mov Disord* **23**, 2129-2170 (2008). https://doi.org:10.1002/mds.22340

24 Tiffin, J. & Asher, E. J. The Purdue Pegboard: norms and studies of reliability and validity. *Journal of Applied Psychology* **32**, 234 (1948). https://doi.org:10.1037/h0061266

25 Agnew, J., Bolla-Wilson, K., Kawas, C. H. & Bleecker, M. L. Purdue pegboard age and sex norms for people 40 years old and older. *Developmental Neuropsychology* **4**, 29-35 (1988).

26 Stiasny-Kolster, K. *et al.* The REM sleep behavior disorder screening questionnaire—a new diagnostic instrument. *Mov Disord* **22**, 2386-2393 (2007).

27 Litvan, I. *et al.* Diagnostic criteria for mild cognitive impairment in Parkinson's Disease: Movement Disorder Society Task Force guidelines. *Mov Disord* **27**, 349-356 (2012). https://doi.org:10.1002/mds.24893

28 Oedekoven, C., Egeri, L., Jessen, F., Wagner, M. & Dodel, R. Subjective cognitive decline in idiopathic Parkinson s disease: A systematic review. *Ageing Res. Rev.* **74**, 101508 (2022). https://doi.org:10.1016/j.arr.2021.101508

29 Ophey, A. *et al.* Cognitive training and promoting a healthy lifestyle for individuals with isolated REM sleep behavior disorder: study protocol of the delayed-start randomized controlled trial CogTrAiL-RBD. *Trials* **25**, 428 (2024). https://doi.org:10.1186/s13063-024-08265-9

30 Jessen, F. *et al.* The characterisation of subjective cognitive decline. *Lancet Neurol.* **19**, 271-278 (2020). https://doi.org:10.1016/S1474-4422(19)30368-0
